# Colonization Ability and Impact on Human Gut Microbiota of Foodborne Microbes From Traditional or Probiotic-Added Fermented Foods: A Systematic Review

**DOI:** 10.3389/fnut.2021.689084

**Published:** 2021-07-29

**Authors:** Marianna Roselli, Fausta Natella, Paola Zinno, Barbara Guantario, Raffaella Canali, Emily Schifano, Maria De Angelis, Olga Nikoloudaki, Marco Gobbetti, Giuditta Perozzi, Chiara Devirgiliis

**Affiliations:** ^1^Research Centre for Food and Nutrition, CREA (Council for Agricultural Research and Economics), Rome, Italy; ^2^Department of Soil, Plant and Food Science, University of Bari Aldo Moro, Bari, Italy; ^3^Faculty of Science and Technology, Free University of Bozen-Bolzano, Bolzano, Italy

**Keywords:** FAIR principles, human studies, foodborne LAB, gut health, food fermentation

## Abstract

A large subset of fermented foods act as vehicles of live environmental microbes, which often contribute food quality assets to the overall diet, such as health-associated microbial metabolites. Foodborne microorganisms also carry the potential to interact with the human gut microbiome *via* the food chain. However, scientific results describing the microbial flow connecting such different microbiomes as well as their impact on human health, are still fragmented. The aim of this systematic review is to provide a knowledge-base about the scientific literature addressing the connection between foodborne and gut microbiomes, as well as to identify gaps where more research is needed to clarify and map gut microorganisms originating from fermented foods, either traditional or added with probiotics, their possible impact on human gut microbiota composition and to which extent foodborne microbes might be able to colonize the gut environment. An additional aim was also to highlight experimental approaches and study designs which could be better standardized to improve comparative analysis of published datasets. Overall, the results presented in this systematic review suggest that a complex interplay between food and gut microbiota is indeed occurring, although the possible mechanisms for this interaction, as well as how it can impact human health, still remain a puzzling picture. Further research employing standardized and trans-disciplinary approaches aimed at understanding how fermented foods can be tailored to positively influence human gut microbiota and, in turn, host health, are therefore of pivotal importance.

## Introduction

A wealth of studies in the past few decades has shown the important contribution of dietary components in health maintenance [reviewed in Papadaki et al. (1), Wallace et al. (2)], with increasing focus on the role of diet in modulating the microbial profile of the gut microbiome (3, 4). Gut microbial composition was increasingly reported to display different characteristics in healthy and diseased conditions, suggesting a potential role in the health/disease balance. However, causal relations and possible mechanisms are not yet conclusive (5). The health-promoting effects of the Mediterranean dietary pattern have recently been related also to the composition of the gut microbial ecosystem, which can be modulated by food components in different ways (6). The best-known example is represented by dietary carbohydrates and fiber, which display selective growth stimulation of specific bacterial groups (prebiotic effect) (7). Fermented foods, on the other hand, which are particularly represented in the diet consumed in Mediterranean countries, as well as in Asian continent, can contribute microbial strains of environmental origin to the host gut microbiota, as they often carry heterogeneous consortia of live bacteria, including species and/or strains that can survive the harsh conditions of the gastrointestinal (GI) tract and find their own niche within the intestinal microbiota of humans. The impact of foodborne microbes on gut microbial composition has been increasingly studied and was proposed as a strategy to improve host health with nutritional interventions (8–11).

The world of fermented foods is quite complex, comprising a broad array of foodstuffs mostly from dairy, meat and vegetable sources, characterized by distinct production processes and consumption frequencies which often reflect local resources and traditional dietary profiles of each country (10, 12). Moreover, some fermented foods contain probiotic strains that can either participate to the fermentative processes or can be added as health-promoting adjuncts. A recent definition of this highly heterogeneous food category, devised by an expert panel of the International Scientific Association for Probiotics and Prebiotics (ISAPP) describes them as “foods made through desired microbial growth and enzymatic conversions of food components” (11). Such definition includes all foods and beverages obtained through fermentation, irrespective of the presence of living microbes within the food matrix at the time of consumption. Although the species and strain composition in the fermenting consortia may greatly differ, as it reflects the microbial ecology of geographically distinct environments, most fermented foods share the presence of high titers of live bacteria. The capacity to deliver this live bacterial component to the human gut has drawn increasing attention, due to the potential interaction with resident gut bacteria and to the contribution of health-associated microbial metabolites and biogenic compounds to the host (13).

Foodborne microbes able to reach the lower gut can transiently merge with the resident microbiota. In this context, the term colonization is referred to the presence of the ingested microbe during the supplementation period, while persistence can be defined as the capacity of foodborne microbes to survive in the gut once the supplementation is dropped (long-lasting colonization). Colonization can be determined in terms of fecal quantification of specific foodborne strains, which essentially reflects the sum of ingested dose, the extent of cell death (occurring mainly in the upper GI tract), and the subsequent replication activity of surviving cells (14). To date, limited scientific evidence is available to demonstrate direct transfer of foodborne microbes from fermented foods to the host gut microbiota, in terms of colonization and persistence. To move from associations to causal relations, and to shed light on the ability of ingested food microbes to colonize and possibly persist in the host gut, well-designed intervention trials and large cohort studies including fermented food consumption are therefore needed (8).

Considering the complex landscape of fermented foods consumed worldwide, a very important aspect to be examined when designing intervention studies is the choice of the specific foods/beverages to include in the intervention, as well as the assessment of other components in the total diet consumed by the subjects which might positively or negatively interfere with the observed outcomes. These aspects are particularly important when the measurable outcomes of the intervention are represented by highly sensitive-omic biomarkers such as the metagenome or metabolome, which can describe the functional profile of the gut microbiome (15). Moreover, to allow a more objective measure of fermented food intake in future studies, specific biomarkers for this food category have recently been proposed (4).

Within this context, despite the existing body of literature covering this field and the publication of recent reviews specifically focusing on food-gut axis (13, 16, 17), a well-defined knowledge base, drawn from a comprehensive analysis of the relevant studies conducted to date, can represent an extremely helpful tool. To contribute to the construction of such a knowledge base, we searched the available literature with a systematic approach to identify all relevant works reporting results on the potential interplay between food and gut microbiomes, including evidence of microbial transfer from food to gut environments. The key issue that we sought to address in our systematic analysis of the available literature, was specifically aimed at compiling the existing evidence of the capacity of ingested microbes from fermented foods to interact with the human host, which represents the essential first step toward the elucidation of their possible long-term impact on human metabolism. We therefore restricted the analysis to fermented foods containing live bacteria and sought to collect experimental evidence of their abilities to: reach the host lower gut in a vital status and alter the pre-existing profile of the resident microbiota; display at least transient colonization capacity in the gut environment, or persistently colonize the host gut for longer periods of time. Our interest was not restricted to fermentative microbes but rather directed to all microbes ingested with the food matrix (excluding potential pathogens), irrespective of their role in the transformation process. This whole foodborne microbial population carries in fact the potential to colonize (and possibly persist) in the human gut.

Within this search, as well as in the subsequent harmonization step of the retrieved results, we considered several different variables: the food matrix (dairy, meat, fish, plant); the origin of foodborne microbes (autochthonous, starter cultures, probiotics); the study design of both intervention and observational studies. We also performed a critical analysis of the experimental protocols and sequencing strategies reported in the different studies, in order to assess their adherence to the FAIR (Findable, Accessible, Interoperable, Reusable) principles and therefore the potential interoperability of different datasets (18).

We believe that the results reported in this systematic review can be further exploited to improve the design of interventions aimed at elucidating the impact of foodborne and environmental microbes on the gut microbiome, and ultimately their contribution to human health.

## Methods

### Search Strategy and Search Terms

The checklist and flowchart of the PRISMA (Preferred Reporting for Systematic Reviews and Meta-Analyses) guidelines were followed for selecting the studies analyzed in this review (19). A preliminary literature screening showed that no recently published reviews analyzed and discussed the impact on human gut microbiota and the colonization potential of foodborne microbes in a comprehensive and systematic way. Therefore, a systematic literature search for peer-reviewed research articles published until March 1, 2021 was carried out on the PubMed and Scopus databases, respectively, according to the following search terms:

(consumption OR supplementation OR ingestion) AND (fermented food OR fermented milk OR dairy OR cheese OR fermented meat OR fermented vegetable OR fermented plant OR fermented fish) AND (microbiota OR microbiome OR microflora) NOT review [Publication Type].

(TITLE-ABS-KEY (consumption OR supplementation OR ingestion) AND TITLE-ABS-KEY (“fermented food” OR “fermented milk” OR dairy OR cheese OR “fermented meat” OR “fermented vegetable” OR “fermented plant” OR “fermented fish”) AND TITLE-ABS-KEY (microbiota OR microbiome OR microflora) AND DOCTYPE (ar).

Two independent researchers performed literature searches in each database.

The inclusion of asterisks in the search string did not result in additional articles to be included in the analysis.

### Inclusion and Exclusion Criteria

The research articles initially admitted for the analysis were published before March 19, 2020. Subsequently, a new search was carried out to update the analysis to March 1, 2021 (see below).

Duplicate articles retrieved by both databases, reviews or systematic reviews, congress proceedings or articles written in languages other than English were excluded. Documents available in the reference list of eligible articles were subsequently screened and selected for analysis, according to the following inclusion and exclusion criteria:

#### Inclusion Criteria

Human intervention studies performed with traditional fermented foods or fermented foods containing live probiotics or synbiotics; human observational studies, provided they took into account a clear association of the fermented food components ingested with the diet with gut microbiota composition; analysis of the ability of foodborne microbe(s) to reach the human intestine and eventually persist in the host gut (colonization); analysis of human gut microbiota composition, assessed by molecular methods.

#### Exclusion Criteria

Animal or cellular studies; supplementation with encapsulated/lyophilized probiotics; supplementation with probiotics carried by a non-fermented food matrix; supplementation with fermented products that did not contain live microbes, such as cooked or pasteurized products (tea/coffee, fermented pasta, bread and bakery products, alcoholic beverages), heat-killed or UV-killed probiotics; supplementation exclusively with prebiotics or bioactive molecules; analysis of gut microbiota assessed only by cultural methods (unless the selected articles provided significant results in terms of colonization of ingested bacterial strains); analysis of gut microbiota indirectly deduced by the detection of microbial metabolites.

Selected articles were collected on the EndNote X9 software (Clarivate Analytics) and Microsoft Office 365 Excel spreadsheets.

## Results

### Literature Search Results and Summary of the Selected Studies

**Figure 1:**
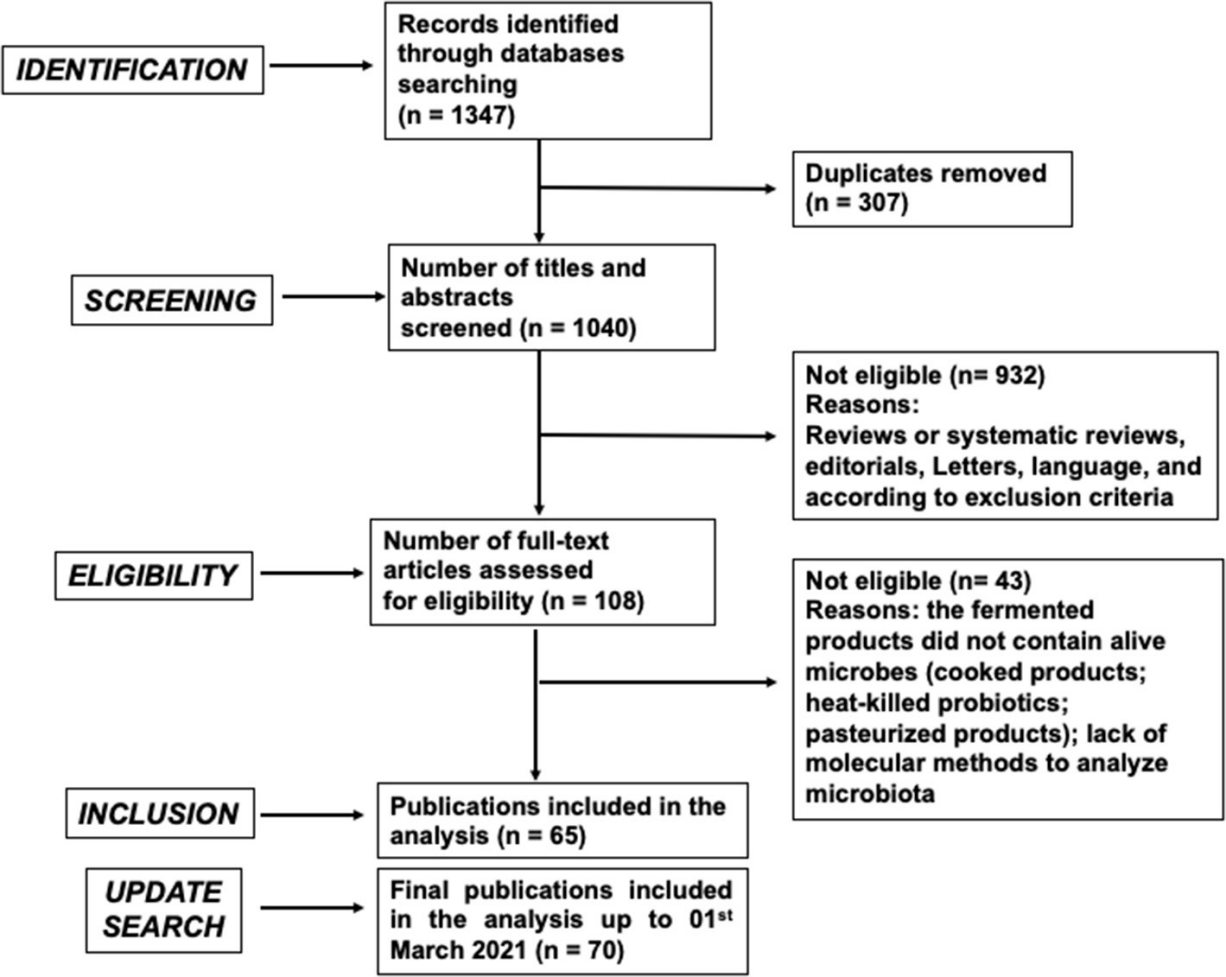
PRISMA flow chart diagram of screening and selection processes applied to identify research articles included in the analysis. Exclusion criteria were the following: Animal or cellular studies; supplementation with encapsulated/lyophilized probiotics; supplementation with probiotics carried by a non-fermented food matrix; supplementation with fermented products that did not contain live microbes, such as cooked or pasteurized products (tea/coffee, fermented pasta, bread and bakery products, alcoholic beverages), heat-killed or UV-killed probiotics; supplementation exclusively with prebiotics or bioactive molecules; analysis of gut microbiota assessed only by cultural methods (unless the selected articles provided significant results in terms of colonization of ingested bacterial strains); analysis of gut microbiota indirectly deduced by the detection of microbial metabolites.

The screening process for documents published up to March 19, 2020 yielded 859 and 488 articles on PubMed and Scopus, respectively, for a total of 1,347 publications. As a result of preliminary analysis, 307 articles were excluded because they proved to be duplicates, yielding a total of 1,040 publications, 932 of which were further excluded on the basis of careful analysis of the titles and abstracts. The majority of the excluded articles focused on animal or *in vitro* models. This procedure resulted in 108 potentially relevant publications. A total of 43 articles were further excluded on the basis of full-text screening. A total of 65 articles were finally selected (Figure 1), and key information was analyzed and included in the results and discussion sections of this manuscript. Subsequently, a new search was carried out with the same criteria as described above, aimed at collecting more recent publications (up to March 1, 2021), leading to the inclusion of 5 additional articles (Figure 1).

Based on the study design, the 70 publications included in the analysis represent 50 intervention and 20 observational studies, which were further sub-divided based on the type of fermented food(s)/probiotic(s)/synbiotic considered, as described in Table 1.

**Table 1:** Classification of the publications included in the analysis based on study design, type of fermented food, and analysis of microbial colonization ability.

**Study design**	**Type of fermented food**	**Publications reporting colonization ability of foodborne microorganisms**
Intervention (50)	Traditional/commercial (11)	3
	Probiotic (34)	22
	Synbiotic (5)	2
Observational (20)	Traditional/commercial (17)	4
	Probiotic (3)	3
	Synbiotic (0)	-

*The number of articles assigned to each category is reported in parenthesis*.

Within each category, a variable number of articles were identified which also dealt with the analysis of gut colonization ability of foodborne microorganisms (Table 1).

For subsequent analysis, the following relevant data were extracted: food characteristics [food matrix origin, presence of probiotic(s)/synbiotic(s)], general study characteristics [food intake evaluation, study design, administered daily amount of food/probiotic(s) and duration of treatment], subjects main characteristics (nationality, health status, gender, age), experimental protocols (methods applied for gut microbiota analysis and microbial groups analyzed, methods applied to evaluate colonization of foodborne microbes), main outcomes (effects on gut microbiota, evaluation of colonization and persistence of foodborne microbes in human gut). Summary of the study characteristics and main findings are shown in Tables 2–**6** and Supplementary Table 1.

**Table 2:** Human intervention studies using traditional or commercial fermented foods.

**Food matrix**	**Administered daily amount and duration of treatment**	**Food intake evaluation**	**Study design**	**Subjects main characteristics**	**Effects on gut microbiota**	**Gut microbiota analysis methods and microbial groups analyzed**	**Colonization evaluation**	**Persistence evaluation**	**Methods of analysis for colonization**	**References**
Yogurt (Control: Milk)	220 g; 24 weeks	Yes	Randomized, parallel and controlled trial (*n* = 92, 48 treatment and 44 control)	Chinese obese females with NAFLD and Metabolic Syndrome; age range 36–66 years	Decreased *Firmicutes, Clostridiales, Blautia, Eubacterium ventriosum, Erysipelotrichaceae, Ruminococcus, Pseudobutyrivibrio, Dialister*; Increased *Phascolartobacterium*	16S rRNA based metagenomics: V4 variable region of the 16S rRNA gene (All bacteria)	No	–	–	(20)
Yogurt (Control: Pasteurized Yogurt)	125 g, containing 1.6 × 10^9^ and 2.5 × 10^10^ CFU *L. delbrueckii* subsp. *bulgaricus* and *S. thermophilus*, respectively; 2 weeks intervention; 2 weeks post-intervention washout	No	Cross-over trial with washout, double blind (*n* = 79, 63 treatment and 16 control)	Spanish healthy young subjects (32 men and 47 women); mean age 23.6 years	Increased LAB and *C. perfringens*; decreased *Bacteroides*. Bacterial changes were not different after the consumption of fresh and heat-treated yogurt	qPCR (*Bacteroides vulgatus; Bacteroides* group; *Clostridium perfringens* and *coccoides* groups); DGGE (Universal bacteria; LAB group)	No	–	–	(21)
Kefir (Control: Milk)	180 ml; 12 weeks	Yes	Randomized, parallel and controlled trial (*n* = 22, 12 treatment and 10 control)	Turkish men and women with Metabolic Syndrome; age range 45–60 years	Increased *Lactobacillus* and *Bifidobacterium* spp. Significant increase only in the relative abundance of *Actinobacteria*. No significant change in the relative abundance of *Bacteroidetes, Proteobacteria, Verrucomicrobia*, or sub-phylum bacterial populations	16S rRNA based metagenomics: V3–V4 variable regions of the 16S rRNA gene (All bacteria)	No	–	–	(22)
Kefir	400 ml, containing 8.0 × 10^12^ CFU viable *Lactobacillus*; 4 weeks	No	Randomized, parallel and controlled trial (*n* = 45, 25 treatment and 20 control)	Turkish patients with Ulcerative Colitis or Crohn's Disease (22 males and 23 females); age range >18 years	Increased *Lactobacillus* levels	qPCR (*Lactobacillus*; *L. kefiri*)	No	–	–	(23)
Parmesan; Parmesan + milk	45 g (Parmesan); 200 ml (milk); 1 week intervention; 1 week post-intervention washout	No	Before-After trial with washout (*n* = 20, 10 Milk and 10 No-Milk, both groups consuming Parmesan cheese)	Italian healthy adults	No differences in the gut microbial profiles of either No-Milk or Milk groups; *Bifidobacterium mongoliense* BMONG18 detected in the feces of all enrolled individuals during the intervention period; decrease of *B. mongoliense* abundance at the end of washout	16S rRNA based metagenomics: V3 variable region of the 16S rRNA gene; whole genome shotgun metagenomics (All bacteria)	Yes	Yes	PCR with strain-specific primers (previously designed based on assembled shotgun metagenomics data)	(24)
Dairy products	nd^a^; 24 weeks	Yes	Randomized, parallel and controlled trial (*n* = 54, 30 treatment and 24 control)	Danish overweight or obese men and women; age range 18–60 years	No significant differences	16S rRNA based metagenomics: V3–V4 variable regions of the 16S rRNA gene (All bacteria)	No	–	–	(25)
Dairy products (semi-skimmed milk, semi-skinned yogurt, buttermilk, low-fat cheese)	High Dairy Diet: 6 portions^b^/day; Low Dairy Diet: 1 portion^b^/day; 6 weeks intervention; 4 weeks post-intervention washout	Yes	Randomized, cross-over trial, with washout (*n* = 46)	Dutch healthy, overweight males and postmenopausal females; age range 45–65 years	During the HDD, significantly higher abundance of the genera *Streptococcus, Leuconostoc*, and *Lactococcus*, and the species *Streptococcus thermophilus, Erysipelatoclostridium ramosum* and *Leuconostoc mesenteroides*; significantly lower abundance of the genera *Faecalibacterium* and *Bilophila*, and the species *Faecalibacterium prausnitzii, Clostridium aldenense, Acetivibrio ethanolgignens, Bilophila wadsworthia* and *Lactococcus lactis*	16S rRNA based metagenomics: V4–V5 variable regions of the 16S rRNA gene (All bacteria)	No	–	–	(26)
Cured meats (salami, prosciutto) and a selection of four cheeses (Blue, Camembert, Caerphilly, Cheddar), as fermented food component of an animal-based diet	*Ad libitum;* 4 days pre-intervention washout; 5 days intervention; 6 days post-intervention washout;	Yes	Cross-over trial with washout (*n* = 11)	American healthy young adults (6 men and 5 women); age range 21–33 years	Increased abundance of bile-tolerant microorganisms (*Alistipes, Bilophila*, and *Bacteroides*) and decreased levels of *Firmicutes* that metabolize dietary plant polysaccharides (*Roseburia, Eubacterium rectale*, and *Ruminococcus bromii*). Foodborne microbes associated with cheese and cured meats (*L. lactis, P. acidilactici*, and *Staphylococcus*) transiently colonized the gut	16S rRNA based metagenomics: V4 variable region of the 16S rRNA gene; RNA-Seq; Fungal ITS sequencing (All microbial groups)	Yes	No	16S rRNA gene amplification and sequencing of cultured microbes from fecal samples and comparison with bacteria associated to cheese or cured meat observed through NGS (species-level)	(27)
Camembert	80 g; 2 weeks pre-intervention washout; 4 weeks intervention; 2 weeks post-intervention washout	No	Before-After trial with washout (*n* = 12)	French healthy adults (6 men and 6 women); age range 19–40 years	Increased *Lactococcus lactis* and *Leuconostoc mesenteroides*. For *Ln. mesenteroides*, persistence was observed 15 days after the end of Camembert consumption. Survival of *Geotrichum candidum* was also detected in stools	qPCR (*Lc. lactis; S. thermophilus; Ln. mesenteroides; Lacticaseibacillus paracasei; L. fermentum; L. plantarum*)	Yes	Yes	qRT-PCR with species-specific primers	(28)
Camembert	80 g; 2 weeks pre-intervention washout; 4 weeks intervention; 2 weeks post-intervention washout	No	Before- After trial with washout (*n* = 12)	French healthy adults (6 men and 6 women); age range 19–40 years	Increased *Enterococcus faecalis*, whose level decreased rapidly after the washout to reach the pre-intervention baseline	qPCR (Bacteria; *E. coli; E. faecalis; E. faecium*)	No	–	–	(29)
Kimchi (Control: Unfermented kimchi)	180 g; 8 weeks	No	Randomized, parallel and controlled trial (*n* = 23, 12 treatment and 11 control)	Korean obese women; age range 30–60 years	Increased *Prevotella* and *Bacteroides* and decreased *Blautia* levels caused by fermented kimchi, but not fresh kimchi	16S rRNA based metagenomics: V1–V3 variable regions of the 16S rRNA gene (All bacteria)	No	–	–	(30)

a *500-kcal–deficit diet that was either high (HD: 1,500 mg calcium/day) or low (LD: 600 mg calcium/day;) in dairy products*,

b *One daily portion consisted of: 250 mL of semi-skimmed milk, 250 mL of buttermilk, 200 g of semi-skimmed yogurt, or 30 g of low-fat cheese*.

### Intervention Studies

#### Traditional or Commercial Fermented Foods

A total of 11 intervention studies dealt with traditional or conventionally commercialized fermented products, not added with claimed probiotic strains (Table 2).

##### Food Matrices and Microbial Composition

The majority of foods were dairies, in particular yogurt (20, 21) and kefir (22, 23), but also cheeses, such as Parmigiano Reggiano (Parmesan) (24) and Camembert (28, 29). Two studies focused on the effect of unspecified dairy products (25, 26), while another study considered a fermented vegetable product, namely kimchi (30). Finally, one study evaluated the impact of a diet based on animal products, in which cured meats and a selection of 4 cheeses represented the fermented food component (27). In some cases, quantification of the live microbial content in the fermented foods was also provided. Microbial composition of dairy products varies among different foods, however, a common feature is represented by the presence of lactic acid bacteria (LAB). While yogurt is characterized by a standardized fermenting microbial ecosystem, composed of *Lactobacillus delbrueckii* ssp. *bulgaricus* and *Streptococcus thermophilus*, other traditional dairy products contain a more complex microbiota, sometimes including also fungi, that can differ both in terms of species composition and microbial load (70). Therefore, the amount of ingested foodborne microbes can significantly vary among the analyzed studies, according to the specific food being considered. Kimchi microbiota, on the other hand, is prevalently composed of *Lactobacillus* and *Leuconostoc* genera (70).

##### Study Design

The studies analyzed in this review were prevalently carried out on adult male and female subjects that were healthy in 6 of the 11 studies (although in one case the healthy subjects were also overweight); obese subjects with or without associated pathologies like metabolic syndrome or Non-Alcoholic-Fatty-Liver-Disease (NAFLD) (*n* = 4); or diseased subjects (*n* = 1). The most widely applied experimental design was represented by randomized, parallel and controlled trial (*n* = 6), followed by before-after trial (*n* = 3) and cross-over trial (*n* = 2). Pre-intervention and/or post-intervention washout was considered in some, but not in all cases. The duration of treatment was extremely variable, ranging from 5 days to 24 weeks. Four of the 11 studies also considered food intake. The amount of daily ingested food ranged from 125 to 400 g for yogurt and kefir; 45 to 80 g for cheese, while kimchi was administered at 180 g/day. In the article by Swarte and colleagues the subjects consumed different amounts of fermented dairy products, expressed as portions according to a Low-Dairy Diet (1 portion/day) or High-Dairy Diet (6 portions/day): one daily portion consisted of: 250 mL semi-skimmed milk, or 250 mL buttermilk, or 200 g semi-skimmed yogurt, or 30 g low-fat cheese (26).

##### Experimental Methodologies

Microbiota composition was prevalently detected by 16S rRNA gene-based Next Generation Sequencing (NGS) (*n* = 7), carried out on fecal samples collected during the intervention period as well as at the end of post-intervention washout, or at baseline, where applicable. Different combinations of the variable regions of 16S rRNA gene were analyzed, with V3–V4 and V4 as the most frequent (*n* = 2 each). Otherwise, gut microbial changes were analyzed by quantitative PCR (qPCR) methods, aimed at detecting bacterial DNA. In these latter cases, specific microbial groups were considered based on the primer sets employed. The most commonly described microbial groups were lactobacilli, even though *Leuconostoc mesenteroides, Lactococcus lactis*, and *S. thermophilus* (28), *Enterococcus* species and *E. coli* (29), *Bacteroides* and *Clostridium* groups (21) were also analyzed in several studies.

##### Effect on Gut Microbiota Composition

Overall, the effect of fermented food ingestion on gut microbiota composition varied among the studies, with the exception of the increased level of the genus *Lactobacillus* that was reported in two articles (22, 23). Two studies reported no significant changes in gut microbiota composition following ingestion of Parmesan (24) or dairy products (25).

##### Colonization and Persistence

The colonization ability of foodborne microbes was analyzed in 3 studies: in particular, the presence of *Bifidobacterium mongoliense* BMONG18 was detected in the feces of all enrolled individuals during the intervention period with Parmesan cheese. This strain had been previously isolated from the cheese used to feed the subjects and characterized at the strain level. Moreover, the authors also showed that the abundance of *B. mongoliense* BMONG18 decreased at the end of post-intervention washout, suggesting that Parmesan consumption is required to maintain long-term persistence of this foodborne strain in the human gut (24). David and coworkers elegantly showed that foodborne microbes associated with cheese and cured meats (*Lc. lactis, Pediococcus acidilactici*, and *Staphylococcus*) transiently colonized the gut during a short-term intervention trial. In this case, the presence of such bacteria was revealed by 16S rRNA gene amplification and sequencing of cultured microbes from fecal samples followed by comparison with bacteria associated to cheese or cured meat detected by NGS (27). Finally, two of the bacterial species characterizing Camembert microbial ecosystem, namely *Lc. lactis* and *Ln. mesenteroides*, were detected in fecal samples of subjects consuming such cheese. In particular, for *Ln. mesenteroides*, persistence was observed 15 days after the end of Camembert consumption. Moreover, survival of the foodborne mold *Geotrichum candidum* was also detected in stool samples by a culture-dependent approach (28).

#### Probiotic or Synbiotic Fermented Foods

Thirty-nine intervention studies focused on the use of fermented foods added with probiotics or synbiotics. Probiotics are defined as “live microorganisms that, when administered in adequate amounts, confer a health benefit on the host” (71). The great majority of probiotic strains belong to the genera *Lactobacillus* and *Bifidobacterium*. Many of the corresponding species have been assigned the Generally Recognized as Safe (GRAS) or the Qualified Presumption of Safety (QPS) status by the Food and Drug Administration (FDA) and the European Food Safety Authority (EFSA), respectively. Lactobacilli, which represent the prominent members of LAB, form a phylogenetically diverse group belonging to the phylum of Firmicutes, and are defined as Gram-positive, catalase-negative, non-spore forming, mostly non-motile, microaerophilic rods. They generally have a fermentative metabolism, with lactic acid as the major end product of carbohydrate fermentation (72). The taxonomy of the genus *Lactobacillus* was recently revised to reclassify species previously belonging to such genus into 25 genera that comprise phylogenetically related micro-organisms. According to this update, the species *casei, paracasei* and *rhamnosus* are now ascribed to *Lacticaseibacillus* genus, while the species *brevis* is now reclassified as *Levilactobacillus brevis* (73). Lactobacilli are important members of the human and animal gut microbiomes and they are highly abundant in several fermented foods. Different probiotic species and strains of lactobacilli, including *L. acidophilus, Lacticaseibacillus casei, Lacticaseibacillus rhamnosus*, and *L. helveticus*, have been extensively employed in animal models and humans to prevent and treat several diseases (74). Moreover, studies with probiotic strains of lactobacilli demonstrated their ability to affect the composition of the host gut microbiota, impacting on its complex ecosystem [(75) and references therein].

The genus *Bifidobacterium* belongs to the phylum Actinobacteria and contains more than 50 species, including several subspecies. Bifidobacteria represent the first microbial colonizers of the newborn intestine, playing a basic role in the development of gut physiology, maturation of the immune system and digestion of dietary components (76, 77). Some *Bifidobacterium* strains are considered probiotic microorganisms thanks to their health-beneficial properties. Unlike lactobacilli, bifidobacteria are not usually detected in traditional fermented foods, but rather they are added as bioactive ingredients in functional foods, mainly dairy products, as well as in food supplements and pharma products where they can be used alone or in combination with other microorganisms (78, 79). Supplementation with bifidobacteria strains has been suggested to exert beneficial effects in some intestinal diseases like antibiotic-associated diarrhea, necrotizing enterocolitis or allergic disorders, such as atopic eczema or rhinitis (80–83). For these reasons, the potentially positive effects of bifidobacteria strains on human gut health are gaining particular interest and an increasing number of experimental studies address the microbiota-mediated impact of bifidobacteria supplementation through fermented food consumption (84).

The studies analyzed in the present Systematic Review were categorized and discussed according to the type of probiotic supplementation. In particular, *Lacticaseibacillus casei* Shirota (LcS) (Table 3) was considered separately from other *Lactobacillus/Lacticaseibacillus* (Table 4) and *Bifidobacterium* strains (Table 5), as well as from synbiotics (Table 6). Indeed, LcS represents, together with *Lacticaseibacillus rhamnosus* GG (LGG), one of the most commonly studied probiotic strains belonging to lactobacilli. However, with respect to LGG, LcS is employed to ferment milk, and supplemented to humans through consumption of the corresponding probiotic drink, while LGG is often supplemented in capsules.

**Table 3:** Human intervention studies using fermented foods containing the probiotic *Lacticaseibacillus casei* Shirota (LcS).

**Food matrix**	**Probiotic**	**Administered daily amount and duration of treatment**	**Food intake evaluation**	**Study design**	**Subjects main characteristics**	**Effects on gut microbiota**	**Gut microbiota analysis methods and microbial groups analyzed**	**Colonization evaluation**	**Persistence evaluation**	**Methods of analysis for colonization**	**References**
Fermented milk	*Lacticaseibacillus casei* Shirota (LcS)	80 g containing 3 × 10^10^ CFU for 4 weeks	No	Cross-over trial, with 4 weeks washout (*n* = 35)	Malaysian children, 16 normal weight and 19 overweight (17 males and 18 females); age range 7–10 years	Increased *Bacteroides ovatus* among the normal weight cohort. Increased *Lachnospira* and *Ruminococcus* in the overweight participants	16S rRNA based metagenomics: V3-V4 variable regions of 16S rRNA gene (All bacteria)	No	No	–	(31)
Fermented milk (Control: acidified milk)	LcS	130 g containing 1.3 × 10^11^ CFU for 3 weeks	No	Double-blind placebo-controlled trial (*n* = 20, 10 treated and 10 placebo) with 1 week post-intervention washout	English healthy adults; age range 23–70 years	Increased lactobacilli, while no effect on total bacteria, *Bacteroides* spp., *Eubacterium rectale*–*C. histolyticum* subgroup, *Atopobium rimae*–*Collinsella*–*Eggerthella lenta* subgroup, and *C. perfringens*/*histolyticum* subgroup or *E. coli* was observed. A stable and relatively high population of LcS (between 6.7 and 7.1 Log_10_ CFU/g feces) was maintained in volunteers during the intervention period. LcS persisted in six volunteers until day 28 at 10^5^ CFU/g feces	Fluorescent *in situ* hybridization (FISH) with molecular probes targeting total bacteria, bifidobacteria, *Bacteroides*, Clostridia (*Clostridium perfringens/ histolyticum* sub-group), *Eubacterium rectale*-*C. histolyticum* sub-group, *Atopobium rimae*-*Collinsella*-*Eggerrthella lenta* sub-group, *Lactobacillus*/*Enterococcus* spp. and *E. coli*	Yes	Yes	LcS was enumerated using Lactitol-LBS Vancomycin (LLV) agar. The identity of putative LcS isolates was confirmed by PFGE	(32)
Fermented milk (Control: milk drink without bacteria, adjusted to match the LcS fermented milk)	LcS (YIT9029 strain)	80 g containing 4 × 10^10^ CFU for 2 months	No	Before/ After trial, with 2 weeks pre-intervention washout (*n* = 10)	Japanese elderly frail subjects (3 males and 7 females); age range 73–93 years	No significant increase of total number bacteria, significant increase of bifidobacteria, total lactobacilli and *L.^a^ casei* subgroup, significant decrease of *Enterobacteriaceae* and *Pseudomonas*. LcS detected in all subjects during ingestion (bacterial count of fecal samples: 10^2^-10^8^ CFU/g)	qRT-PCR using group or species-specific primers (*Clostridium leptum*; *C. coccoides; Bacteroides fragilis; Bifidobacterium; Atopobium* cluster; *Prevotella*; *C. perifringens; C. difficile; L. acidophilus; L.^b^ brevis; L.^a^ casei; L. fermentum; L. fructivorans; L. plantarum; L. reuteri; L. ruminis; L. sakei; Enterobacteriaceae; Enterococcus; Staphylococcus; Pseudomonas*)	Yes	No	qPCR using LcS strain-specific primers	(33)
Fermented milk	LcS (YIT9029 strain)	80 g containing 4 × 10^10^ CFU for 6 months	No	Randomized placebo-controlled double-blind trial, with 3 weeks pre-intervention washout (*n* = 72, 36 treated and 36 placebo; *n* = 20, 10 treated and 10 placebo)	Japanese elderly subjects (19 males and 53 females); age range 72–93 years; staff members (5 males and 15 females); age range (26–49 years)	After 1, 3, and 6 months of ingestion, significant increase of *Bifidobacterium* in the LcS-fermented milk group. Cell number of *C. difficile, C. perfringens, Enterobacteriaceae* lower than placebo. LcS recovered from feces of all subjects at 10^8^ CFU/g during the ingestion period	qRT-PCR (*Bifidobacterium, Lactobacillus, C. difficile, C. perfringens, Enterobacteriaceae, Staphylococcus, Pseudomonas*); YIF-SCAN	Yes	No	qPCR using LcS strain-specific primers	(34)
Fermented milk	LcS	80 g containing 4 × 10^10^ CFU for 16 weeks	No	Randomized controlled trial (*n* = 68, 34 treated and 34 control)	Japanese type 2 diabetes patients with stable glycemic control (49 males and 19 females); age range 30–79 years	Total *Lactobacillus* and *L.^a^ casei, L. gasseri, L. reuteri* subgroups significantly increased. At the end of the study the fecal counts of the *Clostridium coccoides* group and *C. leptum* subgroup significantly higher in the intervention group	RT-qPCR; qPCR; YIF-SCAN	No	No	**–**	(35)
Fermented milk	LcS	80 g containing 1 × 10^10^ CFU for 2 weeks	During the study, daily food intake, concomitant medications, and bowel movement were recorded	Before/After trial, with 2 weeks pre-intervention washout and 2 weeks post-intervention washout (*n* = 21)	Chinese healthy young adults (14 females and 7 males); age range 18–25 years	No effect on gut microbiota composition. LcS detected in all the subjects during the ingestion period (about 10^7^ CFU/g feces); LcS was detected in only 5 subjects at the end of the 14 days post-intervention washout	16S rRNA based metagenomics: V3–V4 variable regions of 16S rRNA gene (All bacteria)	Yes	Yes	ELISA strain-specific	(36)
Fermented milk Yakult	LcS	100 g containing 1 × 10^10^ CFU for 4 weeks	No	Before/After trial, with 2 weeks pre-intervention washout and 2 weeks post-intervention washout (*n* = 62)	Chinese healthy adults (3 groups with different constipation levels); (54 females and 8 males); age range 18–45 years	LcS intervention increased the *Pseudobutyrivibrio* and *Roseburia* abundances in HS (hard stool) and decreased the *Pseudobutyrivibrio* abundance in SS (Soft stool)	16S rRNA based metagenomics: V3–V4 variable regions of 16S rRNA gene (All bacteria)	No	No	–	(37)
Fermented milk	LcS	65 g containing 6.5 × 10^9^ CFU for 6 weeks	No	Controlled trial (*n* = 18, 6 treated and 12 control)	Dutch healthy children (6 males and 12 females); age range 12–18 years	No effect on microbiota composition	Intergenic Spacer Profiling (IS-pro) (All bacteria)	No	No	–	(38)
Fermented milk (Control: acidified milk)	LcS (YIT9029 strain)	65 g containing 1.5 × 10^10^ CFU for 6 months	No	Randomized, double-blind, placebo-controlled parallel trial (*n* = 68, 35 treatment and 33 placebo)	Japanese elderly subjects (57 females and 11 males); mean age 86.3 ± 7.8 years	Significant increase of *Bifidobacterium. Enterobacteriaceae* numbers at the end of ingestion period in both groups were significantly lower than those before the ingestion period, but when the changes between two periods were compared, there were no significant differences between the two groups. No differences in *Clostridium* levels	RT-qPCR for *Bifidobacterium, Enterobacteriaceae, Clostridium perfringens*	No	No	–	(39)
Fermented milk Yakult (Control: acidified milk)	LcS (YIT9029 strain)	100 g containing 1 × 10^11^ CFU for 8 weeks	Daily consumption self-recorded in a diary to check the compliance rate of consumption. Consumption of other fermented milk, yogurt, lactic acid bacteria beverages and probiotic/prebiotic products had to be avoided	Randomized, double-blind, placebo-controlled parallel trial, with 2 weeks pre-intervention washout and 2 weeks post-intervention washout (*n* = 48, 23 treated and 24 control)	Japanese healthy young adults (25 males and 22 females); average age 22.8 ± 0.3 years	Significantly higher numbers of LcS species and significant lower *Bacteroidaceae* in supplemented subjects. LcS detected during the ingestion period but not during post-supplementation period	16S rRNA based metagenomics: V1–V2 variable regions of 16S rRNA gene (All bacteria)	Yes	Yes	Cultivation on Lactitol-LBS Vancomycin (LLV); colony identification through PCR using LcS specific set of primers	(40)
Fermented milk Yakult	LcS	100 g containing 1 × 10^10^ CFU for 14 days	Subjects filled-in a diary on a daily basis on food intake. They were asked to avoid fermented dairy products and eat their habitual diet	Before/After trial, with 14 days pre-intervention washout baseline and 14 days post-intervention washout (*n* = 25)	Chinese healthy adults with BMI 18.5–29.9; (9 males and 16 females); age range 20–40 years	Eleven species had positive correlation with LcS, including *Anaerostipes sp., Bifidobacterium adolescentis, Bacteroides uniformis* and eight uncultured bacterial strains. Fourteen species had negative correlation with LcS, including *Roseburia hominis, R. intestinalis, Clostridium sp., Bacteroides, Lachnospiraceae* and *Blautia wexlerae*, and eight uncultured bacterial strains. LcS was able to survive in the gut but not to persist after supplementation	DGGE (universal bacteria) and sequencing of selected excised bands	Yes	Yes	Culture method combined with ELISA.	(41)
Fermented milk Yakult	LcS (YIT9029 strain)	80 g containing 4 × 10^10^ CFU for 6 months	Not evaluated, but subjects were asked to abstain from ingestion of any product that might contain LcS, other lactic bacteria or probiotics for 3 weeks before the treatment	Before/After trial, with 3 weeks pre-intervention washout and 6 months follow-up (*n* = 23)	Japanese healthy children (14 males and 9 females); age range 4–12 years	During probiotic supplementation, the population levels of *Bifidobacterium* and total *Lactobacillus* increased significantly, while those of *Enterobacteriaceae, Staphylococcus* and *Clostridium perfringens* decreased significantly. The patterns of fecal microbiota and intestinal environment were found to revert to the baseline levels within 6 months following the cessation of probiotic intake. LcS was detected during the ingestion period but not at 6 months post supplementation	RT-qPCR for Total bacteria; *Clostridium coccoides* group; *C. leptum* subgroup; *Bacteroides fragilis* group; *Bifidobacterium, Atopobium* cluster; *Prevotella*; *C. perfringens; C. difficile; Lactobacillus; L. gasseri subgroup; L.^b^ brevis; L.^a^ casei subgroup; L. fermentum; L. fructivorans; L. plantarum* subgroup; *L. reuteri* subgroup; *L. ruminis* subgroup; *L. sakei* subgroup; *Enterobacteriaceae; Enterococcus; Staphylococcus*; MRSA; MRCNS; MSSA; MSCNS	Yes	Yes	qPCR using LcS strain-specific primers	(42)
Fermented milk (Control: milk drink without bacteria, adjusted to match the LcS fermented milk)	LcS	80 g containing 4 × 10^10^ CFU for 4 weeks	Subjects filled in a diary of food consumption. They were asked not to consume any other fermented food or oligosaccharide containing food during the survey period	Randomized, double-blind, placebo-controlled trial, with 2 weeks pre-intervention washout and 2 weeks follow up (*n* = 118, 67 treated and 67 control)	Japanese gastrectomized subjects (42 men and 25 women in each group); mean age 63 ± 11 years	Decreased fecal *Staphylococcus* level. LcS did not colonize the gut	RT-qPCR for total bacteria, *Clostridium coccoides* group; *C. leptum* subgroup; *Bacteroides fragilis* group; *Bifidobacterium*; *Atopobium* cluster; *Prevotella, C. perfringens*; total *Lactobacillus, Enterobacteriaceae, Enterococcus, Staphylococcus* and LcS	Yes	Yes	qPCR using LcS strain-specific primers	(43)

a *Recently renamed Lacticaseibacillus*.

b *Recently renamed Levilactobacillus*.

**Table 4:** Human intervention studies using fermented foods containing probiotic *Lactobacillus and Lacticaseibacillus* strains.

**Food matrix**	**Probiotic**	**Administered daily amount and duration of treatment**	**Food intake evaluation**	**Study design**	**Subjects main characteristics**	**Effects on gut microbiota**	**Gut microbiota analysis methods and microbial groups analyzed**	**Colonization evaluation**	**Persistence evaluation**	**Methods of analysis for colonization**	**Reference**
Fermented milk (Control: acidified milk)	*Lactobacillus*^a^*paracasei* subsp. *paracasei* CNCM I-1518; *L*.^a^ *paracasei* subsp. *paracasei* CNCM I-3689; *L*.^a^ *rhamnosus* CNCM I-3690	1 × 10^9^-1 × 10^11^ CFU (100 g) or 3 × 10^9^-3 × 10^11^ CFU (300 g) for 4 weeks	No	Randomized, double-blind, placebo- controlled trial with 2 weeks pre-intervention washout and 4 weeks post-intervention washout (*n* = 96, 49 treated and 47 placebo)	German healthy adults (44 men and 52 women); age range 18–55 years	Modest modifications. Probiotic strains were detected only during consumption period, and at a significantly higher level in subjects who consumed 300 g/day	16S rRNA based metagenomics: V3–V4 variable regions of the 16S rRNA gene and whole genome shotgun metagenomics (All microbial groups)	Yes	Yes	qPCR with strain-specific primers	(44)
Yogurt	*L. delbrueckii* subsp. *bulgaricus* K98	500 g containing 6.5 × 10^9^ CFU for 2 weeks	No	Before/After trial with 1 week pre-intervention washout (*n* = 20)	Spanish healthy adults (5 men and 15 women); mean age 30 ± 5 years	Increased LAB and *Clostridium perfringens;* decreased *Bacteroides-Prevotella-Porphyromonas*. Viable *L. delbrueckii subsp. bulgaricus* K98 detected in only one volunteer	DGGE for *Lactobacillus genus*. qPCR for *Bacteroides-Prevotella-Porphyromonas* group, the *Clostridium coccoides* group, the *Clostridium perfringens* group, and species-specific assays for *Bacteroides vulgatus*	Yes	No	Isolation on MRS plates, followed by qPCR at species level, and finally identification by 16S rDNA sequencing	(45)
Yogurt	*L. johnsonii* 456	100 g containing 1 × 10^10^ CFU for 7 days	No	Before/After trial with 2 months post-intervention washout (*n* = 11)	American healthy adults	Not analyzed (except viable LAB by colony counting). Recovery of *L. johnsonii* 456 and persistence after washout	nd	Yes	Yes	qPCR with strain-specific primers	(46)
Yogurt (Control: acidified milk)	*L*.^a^*rhamnosus* GG (LGG)	400 g containing 1.2 × 10^9^ CFU for 2 weeks	No	Randomized, double-blind, placebo controlled cross-over trial with 4 weeks pre-intervention washout, 3 weeks washout and 3 weeks post-intervention washout (*n* = 14, 7 treated and 7 placebo)	Swiss healthy men; age range 18–40 years	Increased *S. thermophilus, L. delbrueckii* subp. *bulgaricus* and *Intestinibacter bartlettii;* decreased *Bifidobacteria*. No significant increase of LGG	16S rRNA based metagenomics: V3–V4 variable regions of the 16S rRNA gene (All bacteria)	Yes	Yes	Inferred from NGS data. Species-level	(47)
Fermented milk (Control: pasteurized acidified milk)	LGG and *L. gasseri* TMC0356	110 g containing 1.5 × 10^10^ CFU LGG and 1.1 × 10^9^ CFU *L. gasseri* TMC0356, for 10 weeks	No	Double-blind, randomized, placebo-controlled trial (*n* = 25, 14 treated and 11 placebo)	Japanese adults with Japanese cedar *Cryptomeria japonica* pollinosis (9 men and 16 women); age range 36.9 ± 6.9 years for treated and 36.5 ± 6.1 years for placebo	Decreased *Bacteroidetes/Firmicutes* ratio; decreased *Bacteroides, Parabacteroides, Prevotella* and *Oscillospira;* increased *Collinsella, Lactobacillus, Blautia* and *Ruminococcus*. Detection of LGG at the end of the supplementation	16S rRNA based metagenomics: V4-V5 variable regions of the 16S rRNA gene (All bacteria) and qPCR (LGG, *L.^a^ casei, L. plantarum, L. acidophilus, S. aureus* NUC, and *A. muciniphila*)	Yes (only for LGG)	No	qPCR at species level	(48)
Fermented milk (Control: fermented milk with *Streptococcus thermophilus*)	*L. johnsonii* La1	120 g containing 1 × 10^9^ CFU for 21 days	No	Double-blind placebo-controlled cross-over trial with 21 days pre-intervention washout and 28 days washout (*n* = 22, 11 treated and 11 placebo)	Japanese healthy young women; age range 20–22 years	Increased *Bifidobacterium* and *Lactobacillus;* decreased lecithinase-positive *Clostridium*. Detection of viable *L. johnsonii* La1 during the test, no persistence after the end of supplementation	Culture-dependent method: total bacteria, total anaerobes, total aerobes, *Bacteriodaceae, Bifidobacteria, Clostridia, Lactobacilli, Stroptococci, Staphylococci, Enterobacteriaceae, Bacilli*, Yeasts	Yes	Yes	Isolation on MRS plates, followed by identification of *L. johnsonii* La1 with RAPD-PCR. Strain level	(49)
Fermented milk	*L*.^a^*casei* DN-114 001	300 g containing 3 × 10^8^ CFU, for 10 days	No	Before/After trial with 7 days pre-intervention washout and 10 days post-intervention washout (*n* = 12)	French healthy adults (7 women and 5 men); age range 23–44 years	No significant differences. Recovery of *L*.^a^ *casei* DN-114 001 during supplementation, but not after washout	TTGE (*Bifidobacterium; Lactobacillus–Pediococcus–Leuconostoc–Weissella*)	Yes	Yes	qPCR using *L*.^a^ *paracasei* group-specific primers and Fluorescence *in situ* hybridization (FISH). Species-level	(50)
Fermented milk	*L*.^a^*casei* DN-114 001	300 g containing 3 × 10^8^ CFU for 8 days	No	Before/After trial with 7 days pre-intervention washout and 7 days post-intervention washout (*n* = 7)	French healthy adults (6 men and 1 woman); age range 22–38 years	No significant differences. Recovery of *L*.^a^ *casei* DN-114 001 in feces, where it persisted 3 days after the end of the ingestion	TTGE: *Lactobacillus*; *Pediococcus*; *Leuconostoc*; *Weissella*; FISH: *Atopobium*; *Bacteroides; Prevotella*; *Eubacterium rectale*-*Clostridium coccoides*; Enterobacteria; *Lactobacillus-Enterococcus*; *Streptococcus-Lactococcus*	Yes	Yes	qPCR using *L*.^a^ *paracasei* group-specific primers. Species-level	(51)
Fermented milk (Control: pasteurized yogurt)	*L*.^a^*paracasei* A	100 g containing 1 × 10^10^ CFU for 4 weeks	No	Randomized, parallel and placebo controlled trial with 1 week post-intervention washout (*n* = 26, 13 treated and 13 placebo)	Italian healthy children; age range 12–24 months	Increased lactobacilli. Viable *L*.^a^ *paracasei* A detected after 1 week of consumption	Counts on selective agar media for Clostridia, Bifidobacteria, Total aerobes, Total anaerobes, Enterococci, *Bacteroides*, Enterobacteria, Lactobacilli. DGGE (*Lactobacillus, Bifidobacterium*)	Yes	Yes	Isolation on MRS-Van plates, followed by PCR amplification with strain-specific primers. Strain-level	(52)

a *Recently renamed Lacticaseibacillus*.

**Table 5:** Human intervention studies using fermented foods containing probiotic *Bifidobacterium* strains.

**Food matrix**	**Probiotic**	**Administered daily amount and duration of treatment**	**Food intake evaluation**	**Study design**	**Subjects main characteristics**	**Effects on gut microbiota**	**Gut microbiota analysis methods and microbial groups analyzed**	**Colonization evaluation**	**Persistence evaluation**	**Methods of analysis for colonization**	**References**
Yogurt (Control: yogurt without probiotic)	*Bifidobacterium animalis* subsp. *lactis* MN-Gup	100 g containing 1 × 10^10^ CFU for 4 weeks	No	Randomized, double-blind, placebo-controlled trial with 1 week pre-intervention washout (*n* = 44, 23 treated and 21 placebo)	Chinese healthy adults; age range 18–69 years	Increased *Bifidobacterium, Ruminoccaceae_ UCG-002* and *Ruminoccaceae_UCG-005*; decreased *Roseburia* after intake of MN-Gup yogurt	16S rRNA based metagenomics: V3–V4 variable regions of the 16S rRNA gene (All bacteria)	No	No	–	(53)
Yogurt	*B. animalis* subsp. *lactis* BB-12	250 g containing 2.5 × 10^8^ CFU for 30 days	No	Before/After trial (*n* = 150)	Russian healthy adults; age range 18–40 years	Increased *Bifidobacterium, Streptococcus, Actinobacteria* (*Adlercreutzia equolifaciens* and *Slackia isoflavoniconvertens*) and *Erysipelotrichaceae*	16S rRNA based metagenomics: V4 variable region of the 16S rRNA gene (All bacteria)	No	No	–	(54)
Fermented milk (Control: non-fermented milk product)	*B. animalis* subsp. *lactis* CNCM I-2494 (DN-173 010)	CFU NOT INDICATED 250 g for 14 days	4-day food diary	Randomized, double blind, parallel and controlled trial with 35 days pre-intervention washout (*n* = 106, 53 treated and 53 placebo)	Swedish adults with IBS; age range 18–65 years	No variation of *Bacteroides, Prevotella, Ruminococcaceae, Blautia, Faecalibacterium and Bifidobacterium*	16S rRNA based metagenomics: V3–V4 variable regions of the 16S rRNA gene (All bacteria)	No	No	–	(55)
Yogurt	*B. longum* strain BB536	200 g containing 4 × 10^9^ CFU for 14 or 20 days	Yes	Randomized, parallel and controlled trial with 7 days pre-intervention washout (*n* = 33, 22 treated and 11 control)	Japanese healthy adults (10 men and 23 women); age range 20–50 years	No significant modification of gut microbiota composition	16S rRNA based metagenomics: V3–V4 variable regions of the 16S rRNA gene (All bacteria)	No	No	–	(56)
Fermented milk (Control: acidified milk)	*B. animali*s subsp. *lactis* CNCM I-2494 (DN-173 010)	250 g containing 2.5 × 10^10^ CFU for 4 weeks	No	Randomized, double blind, parallel and placebo controlled trial with 11 days pre-intervention washout (*n* = 28, 13 treated and 15 placebo)	European women with IBS; age range 20–69	Decreased pathobiont *Bilophila wadsworthia;* Increased *Clostridiales*. Species *B. animalis subsp. lactis* detected in stools	Whole genome shotgun metagenomics (All microbial groups)	Yes	No	Inferred from whole genome shotgun metagenomics (species-level metagenomic approach)	(57)
Yogurt (Control: UHT milk)	*B. longum* BB536	160 g containing 1.12 × 10^8^ CFU for 8 weeks	No	Randomized, parallel and controlled trial with 4 weeks pre-intervention washout and 12 weeks post-intervention washout (*n* = 32, 16 treated and 16 control)	Japanese healthy adults positive to enterotoxigenic *B. fragilis* (ETBF); mean age 39.85 years	Significant decrease in the cell number of ETBF	qPCR specific to ETBF strains	No	No	–	(58)
Fermented milk	*B. animalis* subsp. *lactis* CNCM I-2494 (DN-173 010)	230 g containing 1.1 × 10^10^ CFU for 7 weeks	No	Before/After trial with 4 weeks pre-intervention washout and 4 weeks post-intervention washout (*n* = 7)	American healthy adult women monozygotic twin pairs; age range 21–32 years	No detectable perturbation in fecal bacterial species composition. *B. animalis* subsp. *lactis* CNCM I-2494 transiently colonized the gut	16S rRNA based metagenomics: V2 variable region of the 16S rRNA gene, whole genome shotgun metagenomics (All microbial groups)	Yes	Yes	qPCR strain-specific	(59)
Yogurt (Control: yogurt without probiotic)	*B. animalis* subsp. *lactis* LKM512	100 g containing 5.2 × 10^9^ CFU for 2 weeks	No	Cross-over trial with 1 week pre-intervention washout and 2 weeks washout (*n* = 11)	Japanese hospitalized elderly patients (5 women and 6 men); mean age 76.9 years	Increase of *B. animalis* subsp. *lactis*; decrease of *Lactobacillus* spp.	T-RFLP analysis (350 colonic bacterial species and phylotypes)	Yes	Yes	qPCR specie-specific (*B. animalis* subsp. *lactis, B. adolescentis, Enterococcus, Lactobacillus* spp and total bacteria)	(60)
Yogurt	*B. animalis* subsp. *lactis* DN-173 010 (CNCM I-2494)	125 g containing 3.8 × 10^10^ CFU for 2 weeks	No	Before/After trial with 1 week pre-intervention washout and 1 week post-intervention washout (*n* = 11)	Chinese lactose-intolerant subjects (5 men and 6 women); age range 23–54 years	Increased total bacteria and *Eubacterium rectale*/*Clostridium coccoides* group in feces. No variation in the composition of the predominant bacterial groups. No colonization was detected, nor persistence	FISH (total bacteria, *Bacteroides/Prevotella*; *Eubacterium rectale/Clostridium coccoides* group; *Eubacterium; Ruminococcus* group; *Bifidobacterium*)	Yes	Yes	DGGE (*Bifidobacterium* markers: *B. adolescentis, B. bifidum, B. breve, B. dentum, B. longum, B. pseudolongum, B. animalis*)	(61)
Yogurt (Control: lyophilized probiotic)	*B. animalis* subsp. *lactis* DN-173 010 (CNCM I-2494)	375 g containing 6.6 × 10^10^ CFU for 1 week	No	Randomized, parallel and controlled trial with 10 days pre-intervention washout and 10 days post-intervention washout (*n* = 12, 6 fermented product treated and 6 lyophilized strain treated)	French healthy adults (8 women and 4 men); age range 24–46 years	No major modification in the dominant members of the fecal microbiota. *B. animalis* subsp. *lactis* DN-173 010 survived after 1 week administration but did not persist in all subjects after 10-day follow up	FISH (*Atopobium; Bacteroides/Prevotella; Bifidobacterium genus; Eubacterium rectale-C. coccoides; F. prausnitzii; Lactobacillus-Enterococcus; B. animalis, B. lactis, B. gallicum, B. pseudolongum*)	Yes	Yes	Colony immunoblotting, TTGE and FISH	(62)
Yogurt (Control: yogurt without probiotic)	*B. animalis* subsp. *lactis* LKM512	100 g containing 5.2 × 10^9^ CFU for 4 weeks	No	Double-blind, placebo-controlled, cross-over trial with 4 weeks washout (*n* = 10)	Japanese adults diagnosed with moderate atopic dermatitis (4 men and 6 women); mean age 22.1 years	Increase of bacterial species of *Bifidobacterium* and *Clostridium* clusters	T-RFLP analysis (350 colonic bacterial species and phylotypes)	No	No	–	(63)
Fermented milk	*B. animalis* subsp. *lactis* V9; *Lactobacillus*^a^ *casei* Zhang^b^ (LCZ)	200 g containing 3.6 × 10^10^ CFU V9 and 1 × 10^9^ CFU LCZ, for 4 weeks	No	Before/After trial (*n* = 24)	Chinese healthy adults (5 men and 19 women), age range 29.04 ± 5.58 years	Increased *B. animalis;* decreased *B. catenulatum, B. breve, B. bifidum*, and *B. longum*	Single molecule, real-time (SMRT) sequencing technology with *Bifidobacterium*-specific primers (*Bifidobacterium* spp.)	No	No	–	(64)

a *Recently renamed Lacticaseibacillus*.

b *Although the fermented milk contained both Bifidobacterium and Lactobacillus probiotic strains, this paper was included in the Bifidobacterium-containing products due to the higher microbial titer of Bifidobacterium strain*.

**Table 6:** Human intervention studies using fermented foods containing synbiotics.

**Food matrix**	**Probiotic/s and prebiotic/s**	**Administered daily amount and duration of treatment**	**Food intake evaluation**	**Study design**	**Subjects main characteristics**	**Effects on gut microbiota**	**Gut microbiota analysis methods and microbial groups analyzed**	**Colonization evaluation**	**Persistence evaluation**	**Methods of analysis for colonization**	**References**
Fermented milk (Control: fermented milk without probiotics and prebiotics)	*Lactobacillus*^a^*rhamnosus* IMC 501® and *L*.^a^ *paracasei* IMC 502® + oat bran fiber	200 g containing 1 × 10^9^ CFU for each strain + 8 g oat bran fiber, for 4 weeks	No	Double blind randomized placebo controlled trial (*n* = 10, 5 treated and 5 placebo)	Italian healthy adults (3 men and 7 women); age range 20–45 years	Lactobacilli and Bifidobacteria increased, no differences for the other groups	qPCR for *Lactobacillus* spp., *Bifidobacterium* spp., *Enterobacteriaceae, Clostridium coccoides*-*Eubacterium rectale* group, *Staphylococcus* spp., *Bacteroides*-*Prevotella*-*Porphyromonas* spp	No	–	–	(65)
Fermented milk (Control: heat-treated fermented milk without probiotics and fibers)	*L. acidophilus* La-5 and *Bifidobacterium animalis* subsp. *lactis* BB-12 + dietary fiber Beneo Orafti Synergy 1 (90% inulin, 10% oligofructose)	180 g containing on average 6.5 × 10^9^ CFU La-5 and 9 × 10^9^ CFU BB-12 + 7.2 g dietary fiber, for 4 weeks	No	Double blind randomized placebo-controlled multicentric trial with 2 weeks pre-intervention washout and 1 week post-intervention washout (*n* = 30, 11 treated and 19 placebo)	Slovenian and Croatian adults with IBS; age range 18–65 years	The abundance or proportion of *Enterobacteriaceae, Lactobacillus, Bifidobacterium*, or all bacteria was not affected by consumption of the synbiotic or placebo, except for a transient increase of *Streptococcus thermophilus* Both La-5 and BB-12 strains colonized, but vitality of La-5 was not confirmed. Persistence was not found	qPCR for *Enterobacteriaceae, Bifidobacterium* genus, *Lactobacillus* group, all bacteria 16S rRNA based metagenomics: V4 variable region of 16S rRNA gene (All bacteria)	Yes	Yes	Strain and subspecies-specific qPCR for La-5 and *B. animalis* subsp. *lactis*. RAPD-PCR profiling of cultured colonies for La-5 and BB-12 vitality	(66)
Yogurt	*L*.^a^*rhamnosus* GR-1 + *Moringa oleifera* leaves	250 g containing 1 × 10^10^ CFU GR-1 + 4.3 g dried ground *Moringa oleifera* leaves. 6 days/week from time of recruitment (last 2 trimesters) until exiting the study (1 week to 1 month postpartum). Mean of treatment duration: 88 days ± 31	48 h dietary recall	Open label trial (n=56, 26 treated and 30 control) Longitudinal over pregnancy and after delivery	Tanzanian pregnant women; age range 18–40 years	Yogurt consumption had no effect on the mother's microbiota (but increased the relative abundance of *Bifidobacterium* and decreased *Enterobacteriaceae* in the newborn feces). No clear trend was detected when comparing probiotic to control groups	16S rRNA based metagenomics: V4 variable region of 16S rRNA gene (All bacteria)	No	–	–	(67)
Yogurt GRANAROLO ViviVivo (Control: pasteurized yogurt without probiotics and prebiotics)	*L*.^a^*rhamnosus* GG (LGG) + FOS-Actilight	250 g containing approximately 2.5 × 10^9^ CFU LGG + 6 g FOS, for 4 weeks	No	Adults: double blind randomized placebo-controlled trial with 2 weeks post-intervention washout (*n* = 38, 21 treated and 17 placebo). Elderly: Before-After trial with 2 weeks post-intervention washout (*n* = 12)	Two independent studies: Italian healthy adults (19 men and 19 women); age range 35–60 years, and elderly women with constipation, age range 76–90 years	No significant changes in bifidobacteria counts and *Bifidobacterium* spp. abundance were observed after supplementation, both in elderly and treated adults. LGG was able to colonize and persisted after washout in adults, whereas it only partially colonized and did not persist in elderly	Semi-quantitative PCR for total Bifidobacteria and for the 4 *Bifidobacterium* species: *B. bifidum, B. longum, B. adolescentis* and *B. catenulatum*	Yes	Yes	Semi-quantitative LGG strain-specific PCR	(68)
Fermented milk	*L. acidophilus* CSG, *L*.^b^ *brevis* HY7401, *Bifidobacterium longum* HY8001, *L*.^a^ *casei* HY2782 + fiber + lactulose	140 g containing 2.8 × 10^10^ CFU *Lactobacillus* + 6 g dietary fiber + 2 g lactulose, for 3 weeks	No	Before-After trial with 3 weeks post-intervention washout (*n* = 6)	Korean healthy adult women; age range 20-24 years	Relative abundance of Bacteroidetes (*Bacteroidaceae* and *Prevotellaceae*) increased during the treatment period and decreased during washout. Firmicutes (*Ruminococcaceae* and *Lachnospiraceae*) changed in the opposite way	16S rRNA based metagenomics: V1–V3 variable region of 16S rRNA gene (All bacteria)	No	–	–	(69)

a *Recently renamed Lacticaseibacillus*.

b *Recently renamed Levilactobacillus*.

#### Lacticaseibacillus casei Shirota

A total of 13 publications considered fermented foods containing the probiotic *Lacticaseibacillus casei* Shirota (LcS), previously known as *Lactobacillus casei* Shirota (Table 3). In all studies, subjects were supplemented with a fermented milk containing LcS YIT9029, represented in most studies by the commercial drink Yakult, which includes exclusively LcS as fermenting microorganism.

##### Study Design

The studies were carried out on healthy (*n* = 5), diabetic (*n* = 1), and gastrectomized (*n* = 1) adults, or elderly frail subjects (*n* = 3) and healthy or overweight children (*n* = 3). The duration of supplementation was 2 weeks up to 6 months, and the amount of supplemented LcS ranged from a minimum of 6.5 × 10^9^ Colony Forming Units (CFU)/day to a maximum of 1 × 10^11^ CFU/day. The amount of daily ingested fermented foods used in supplementation was 65–130 g. All articles examined microbiota modifications following supplementation, although some of the studies reported comparison between the initial and final time points in each subject (*n* = 5), while others compared the results in supplemented subjects with those obtained in a placebo group (placebo controlled, *n* = 6), or a control group (*n* = 1); one study applied a cross-over trial (*n* = 1). Pre- and/or post-intervention washout was applied in the majority of the studies (*n* = 10). Overall food intake was assessed in only 4 studies.

##### Experimental Methodologies

Six out of the 13 articles employed quantitative Reverse Transcriptase-PCR (qRT-PCR) to analyze bacterial mRNA, or qPCR to analyze bacterial DNA, using group or species-specific primers, aimed at studying changes of specific bacterial groups in the gut. In particular, a highly sensitive microbial analytical system, the Yakult Intestinal Flora-SCAN (YIF-SCAN), based on qRT-PCR to selectively quantify the intestinal bacteria, was used in some studies.

The most commonly considered groups were lactobacilli, *Bacteroides, Clostridium, Enterococcus, Staphylococcus*, and *Enterobacteriaceae*. One of the works applied Denaturing Gradient Gel Electrophoresis (DGGE) analysis, while only 4 studies examined the whole microbiome by NGS performed on V3–V4 (*n* = 3) or V1–V3 (*n* = 1) variable regions of 16S rRNA gene.

##### Effect on Gut Microbiota Composition

Comparative analysis of the overall effect of supplementation with LcS-containing fermented milk on gut microbiome composition showed an overall increase of all *Lactobacillus* species in 4 studies (32, 33, 35, 42), an increase in bifidobacteria in 5 studies (33, 34, 39, 41, 42) and a decrease of *Enterobacteriaceae* and *Staphylococcus* in 4 (33, 34, 39, 42) and 2 studies (42, 43), respectively. As regard to other bacterial group modifications, heterogeneous results were reported (31, 37). Finally, no significant changes in gut microbiota composition were observed in two studies (36, 38).

##### Colonization and Persistence

Eight studies evaluated LcS colonization at the end of supplementation, while 6 also considered its persistence following the supplementation period. Almost all studies demonstrated that LcS DNA or RNA could be detected in the stool samples collected during and at the end of the supplementation period. Moreover, some of the studies revealed the presence of live LcS by culture-dependent methods followed by further, specific molecular analysis to confirm LcS identity (32, 40, 41). Interestingly, all studies where persistence was evaluated (*n* = 6) (32, 36, 40–43) demonstrated that no LcS could be detected in volunteer stool samples when tested at various time points (ranging from 1 week to 6 months of post-intervention washout) after completion of the study, except in very rare cases (32, 36), further suggesting that regular consumption is required for persistence of ingested probiotic strains in the gut.

#### Other Lactobacilli

A total of 9 studies focused on fermented foods containing probiotic *Lactobacillus/ Lacticaseibacillus* strains, belonging to the following species: *L. casei, L. paracasei, L. johnsonii, L. rhamnosus, L. gasseri* and *L. delbrueckii* ssp. *bulgaricus* (Table 4). As stated above, the species *casei, paracasei* and *rhamnosus* are now ascribed to *Lacticaseibacillus* genus.

Food types were mostly represented by fermented milk (*n* = 6) or yogurt (*n* = 3).

##### Study Design

While 8 studies were performed on healthy subjects, one article analyzed the modulation of gut microbiota in individuals affected by the allergic disease Japanese cedar pollinosis (JCP) (48). Among the 9 analyzed studies, 8 interventions were performed on adult subjects, while Marzotto et al. conducted a randomized placebo-controlled trial on 12–24 months aged children (52). The methodologies of the trials varied between studies. Four of them applied the before-after trial experimental design, 3 studies performed a randomized, parallel and controlled trial, while 2 studies were designed as a cross-over trial. In most studies, pre-intervention and/or post-intervention washout was included (*n* = 8). The duration of treatments varied among studies, ranging from 1 to 10 weeks. Adequate description of the treatment was provided in all the analyzed publications, but none of them reported overall food intake assessment. The amount of ingested probiotic strains varied among the different studies (ranging between 1 × 10^8^ and 1 × 10^11^ CFU/day), while the amount of daily ingested fermented food ranged between 100 and 500 g.

##### Experimental Methodologies

The analysis of gut microbiota composition was performed using different methods, which were often applied in combination. Three studies analyzed microbiota variations through 16S rRNA based or whole genome shotgun metagenomics (44, 47, 48) allowing detection of all microbial groups, while qPCR was used to evaluate the presence of specific microbial groups (45, 48). NGS was performed on V3–V4 (*n* = 2) or V4–V5 (*n* = 1) variable regions of the 16S rRNA gene. Moreover, Temporal Temperature Gradient Gel Electrophoresis (TTGE) (*n* = 2) and DGGE (*n* = 2) were performed to identify the presence of specific genera. In addition to the TTGE technique, Fluorescence *in situ* hybridization (FISH) analysis was applied in one case (51). Two studies, although applying only culture-dependent methods (46, 49), were included in the analysis since they evaluated the colonization ability of the probiotic strains contained in fermented foods. However, one of the two articles reported the effect on gut microbiota exclusively in terms of viable LAB counts (46).

##### Effect on Gut Microbiota Composition

Despite the reported differences in the duration of treatments or in the detection techniques employed, 6 studies highlighted an increase of LAB genera, in particular *Lactobacillus* spp. (45–49, 52). The increase was at times accompanied by reduced numbers of *Bacteroides* or *Clostridium* spp. (45, 48, 49, 52). On the other hand, conflicting results were obtained at the level of gut bifidobacterial species, which were reported to increase (49), decrease (47), or remain unaffected (52). One study reported that, while gut microbiota structure was modestly modified after consumption, a few genera corresponding to *Lactobacillus, Holdemania*, and Clostridiales were differentially affected in response to different doses of ingested probiotic food (44). Finally, 2 studies reported no significant changes in the overall composition of gut microbiota following fermented milk ingestion (50, 51).

##### Colonization and Persistence

All the 9 studies analyzed the colonization ability of probiotic bacteria, through qPCR analysis (*n* = 6), random amplification of polymorphic DNA (RAPD)-PCR (*n* = 1), PCR amplification (*n* = 1) or by inference from NGS data (*n* = 1). Three studies performed isolation of probiotic colonies prior to molecular characterization (45, 49, 52), enabling the detection of viable cells. Seven out of 9 studies evaluated also bacterial persistence after the supplementation phase. The results of colonization analyses highlighted the ability of all probiotic strains to transiently colonize the human gut, with the only exception of the LGG strain in one of the trials (47). Moreover, the strains *L. johnsonii* 456, *Lacticaseibacillus casei* DN-114 001 and *Lacticaseibacillus paracasei* A were able to survive in the gut after the washout period, lasting for 3 to 10 days after the washout period (46, 50–52). It must be pointed out that in the subjects supplemented with *L. johnsonii* 456, the strain was detected at higher levels over time, but this difference was not significant, probably due to the high variability and low number of enrolled participants (46). On the other hand, *L. johnsonii* La1 was recovered in the feces at the end of the test phase, while it was no longer detectable during the post-intervention washout (49). Finally, viable counts of the *L. delbrueckii* ssp. *bulgaricus* K88 strain were observed in one study, but only in one out of 20 volunteers (45).

#### Bifidobacteria

Twelve intervention studies included in the analysis were focused on fermented foods containing probiotic bifidobacterial strains, alone or in combination with other probiotic strains (Table 5). The food matrix was of dairy origin in all cases, in particular fermented milk (*n* = 4) and yogurt (*n* = 8). Strains belonging to *B. animalis* subsp. *lactis* were employed in 10 studies, while *B. longum* BB536 strain was used in 2 studies. It should be mentioned that one specific strain of *B. animalis* subsp. *lactis* is referred as CNCM I-2494 or DN-173 010 in different studies, although representing the same strain (85).

##### Study Design

Five works analyzed adult subjects presenting diseased conditions such as irritable bowel syndrome (IBS) (55, 57), atopic dermatitis (AD) (63), and lactose intolerance (61), while only one of them studied hospitalized elderly patients (60). Six studies were conducted on healthy subjects, while 1 article was focused on healthy subjects found to be enterotoxigenic *Bacteroides fragilis* (ETBF) carriers (58). The great majority of these studies applied double blind randomized, parallel and controlled trial (*n* = 6) as experimental design; the before-after trial design was applied in 4 studies and a cross-over design was reported in 2 articles. Pre-intervention and/or post-intervention washout was carried out in 8 studies, while daily food intake during the intervention period was considered in only 2 works (55, 56). Strains belonging to *B. animalis* subsp. *lactis* were used at a concentration ranging between 1 × 10^8^ and 1 × 10^10^ CFU/day, with an intervention period ranging from 1 to 8 weeks; while the *B. longum* BB536 strain was used at a concentration of 1 × 10^8^-10^9^ CFU/day for 2 or 8 weeks. The amount of ingested food ranged between 100 and 375 g/day.

##### Experimental Methodologies

Different approaches were applied to analyze variations in gut microbiota composition, with whole genome shotgun metagenomics and 16S rRNA based metagenomics employed in 3 and 5 studie, respectively, to identify all bacterial groups; qPCR was performed in 1 study while terminal restriction fragment length polymorphism (T-RFLP) and FISH assays were applied in 5 studies to detect specific bacterial groups. One study employed the Single Molecule, Real-time (SMRT) sequencing technology with *Bifidobacterium*-specific primers to exclusively detect the gut bifidobacterial population (64). 16S rRNA based metagenomics was prevalently performed on V3-V4 variable regions of the 16S rRNA gene (*n* = 3), followed by V2 (*n* = 1) or V4 (*n* = 1) (Table 5).

##### Effect on Gut Microbiota Composition

Regarding the impact of supplementation on gut microbiota composition, 5 publications reported no significant effect (55, 56, 59, 61, 62). On the contrary, overall increase in gut *Bifidobacterium* level was observed in 4 studies (53, 54, 60, 63), while alteration of other microbial groups varied among studies, often depending on the health condition of the subjects. Interestingly, significant effects were observed in unhealthy subjects or when subjects were clustered into subgroups based on their baseline microbiota composition (54, 55), suggesting the importance of including clustering approaches in the selection of volunteers for intervention studies. In particular, the intake of a probiotic fermented milk containing the CNCM I-2494 strain of *B. animalis* subsp. *lactis* appeared to exert major beneficial effects in high-H_2_ producing IBS patients with higher metabolic potential due to increased *Prevotella*/*Bacteroides* ratio, as compared to low H_2_ producers (55). Furthermore, Veiga et al. investigated variations in gut microbiota profiles in IBS subjects supplemented with fermented milk containing the same *B. animalis* strain and observed a decreased amount of the pathobiont *Bilophila wadsworthia*, accompanied by increased levels of butyrate-producing bacteria belonging to the Clostridiales microbial group (57). Another interesting clustering effect was observed by Bai et al.: in this study, the gut bifidobacterial profiles prior to intervention could be assigned to five distinct enterotype-like clusters, each one characterized by one or two dominant bifidobacterial species. These clusters were differentially affected by the fermented milk supplementation (64). In another study, the intake of yogurt containing *B. animalis* subsp. *lactis* LKM512 appeared to induce specific alterations in the bacterial species and phylotypes of *Bifidobacterium* and *Clostridium* clusters in healthy subjects (63), while it appeared to increase the overall biodiversity of the intestinal microbiota, and to decrease the levels of *Lactobacillus* spp. (as compared to the placebo group) when administered to hospitalized elderly volunteers (60). Although the impact of probiotic supplementation on the overall gut microbiota composition was not analyzed, significant effect of the *B. longum* BB536 strain was observed in reducing fecal levels of ETBF in healthy subjects testing positive to such strains (58).

##### Colonization and Persistence

The colonization capacity of probiotic bifidobacterial strains was investigated in 5 of the selected articles, using different molecular approaches such as whole genome shotgun metagenomics (57), qPCR (59, 60), DGGE (61), and colony immunoblotting, TTGE and FISH (62). Although most of the studies (*n* = 4) demonstrated a transient probiotic strain colonization of the human gut, the work by He et al., showed that the *B. animalis* subsp. *lactis* strain DN-173 010 was apparently unable to colonize, nor to persist in the gut (61).

#### Synbiotics

Five intervention studies evaluated the effects of synbiotic fermented food administration (Table 6). The “synbiotic” definition has been recently updated to “a mixture comprising live microorganisms and substrate(s) selectively utilized by host microorganisms that confers a health benefit on the host” (86). The association is believed to be more efficient in terms of gut health and function, as compared to probiotics and prebiotics alone. The underlying concept is that selected prebiotic component(s) introduced in the gastrointestinal tract should selectively stimulate the growth and/or activate metabolism of the beneficial component in the resident gut microbiota or improve survival of probiotic microorganisms in the gastrointestinal tract, thus conferring more stable beneficial effects to host health than either one of the two treatments alone. An ideal synbiotic supplement should contain appropriate single or multi strain probiotic(s) and a suitable mixture of prebiotics, where the latter both selectively favors growth and survival of the former as well as multiplication of other endogenous beneficial bacteria in the gut. Based on this approach, synbiotic formulations often contain a mixture of lactobacilli and bifidobacteria strains, as multistrain preparations may exert improved functionality over single strains. Three out of the 5 studies on synbiotics analyzed in this systematic review used multistrain mixtures, while the other 2 used two different single strains of *Lacticaseibacillus rhamnosus* as probiotics. The food matrix employed as vector for synbiotics was always represented by dairies.

##### Study Design

The majority of the analyzed studies (*n* = 3) applied double blind randomized, parallel and controlled trial as experimental design; 1 article reported on a before-after trial and another study used an open label trial. A post-intervention washout period was considered in 3 studies, one of which also applied a pre-intervention washout (66). Daily food intake during the intervention was recorded in only 1 study. While 4 of the 5 studies were quite homogeneous in terms of the duration of treatment [4 weeks in 3 of the studies and 3 weeks in the study by Unno et. al. (69)], the article by Bisanz et al. (67) reported a somewhat different supplementation program: pregnant women were in fact supplemented during the last two trimesters of pregnancy until 1 week to 1 month postpartum. The study subjects were therefore treated for ~12 weeks (88 days), but the length of supplementation was not the same for all participants. The amount of fermented products consumed was 140–250 g/day, containing between 2 × 10^7^ and 1 × 10^11^ CFU of probiotics included in the synbiotic preparations.

##### Experimental Methodologies

The analytical methods employed to evaluate gut microbiota composition were based on qPCR (*n* = 2), 16S rRNA based metagenomics (*n* = 2) or both (*n* = 1). 16S rRNA based metagenomics was performed on V1–V3 or V4 variable regions of 16S rRNA gene (Table 6).

##### Effect on Gut Microbiota Composition

Concerning the observed results, while Granata et al. detected no significant changes in Bifidobacteria counts (68), Coman et al. reported an increase in both bifidobacteria and lactobacilli (65), hypothesizing that the observed increase in bifidobacteria could be attributed to decreased intestinal pH due to metabolites produced by the probiotic strains. On the other hand, Unno et al. reported increased *Bacteroidaceae* and *Prevotellaceae* and decreased *Ruminococcaceae* and *Lachnospiraceae* following synbiotic milk ingestion (69). Two studies reported no differences in microbiota composition between the synbiotic and the placebo group (66, 67).

Only some of the cited studies briefly mentioned a possible contribution of the prebiotic component to the observed changes in microbiota profiles. Granata et al. reported that “the administration of FOS did not induce a significant change in bifidobacteria” (68), while Coman et al. state that “the presence of oat bran could determine the increase of bifidobacteria due to its known bifidogenic effect” (65). Indeed, dietary fiber can be a prebiotic in one host but not in another (87).

##### Colonization and Persistence

Only 2 studies analyzed the colonization and persistence of the probiotic strains, demonstrating their presence in the feces of the subjects at the end of supplementation period (66, 68). Interestingly, Granata et al. highlighted a difference in the colonization/persistence capability between adult and elderly subjects: while the ability of the LGG strain to survive in the gastrointestinal tract was limited in the elderly, the same strain was able to colonize the gut of almost all treated adult subjects, persisting at least partially during the post-intervention washout (68).

### Observational Studies

#### Traditional or Commercial Fermented Foods

Twenty observational studies were included in this analysis (Supplementary Table 1). Among them, 17 articles reported studies with traditional and fermented foods consumed as part of the habitual diet.

##### Food Matrices and Microbial Composition

The majority of fermented foods were represented by dairy products (88–90), especially fermented milk (91–93), yogurt (94–96), and different cheese varieties (97). Other fermented foods included plant and vegetable based (15, 98), fermented rice (99), and diverse home-made (100) and local (101–103) fermented products. The amount of ingested fermented foods varied between 135 and 400 g/day, although not all articles reported this information, and when reported, the information was based on food frequency questionnaire (FFQ). Finally, quantification of live microbes in food was provided only in a few cases.

##### Study Design

The studies were carried out on individuals of both genders, belonging to different age groups. Only two studies were conducted on diseased subjects, affected by autism spectrum disorder (ASD) (93) or suffering from major depressive disorder (MDD) (92), while the other 18 involved healthy individuals. Demographic factors were considered in all articles, with 4 of them considering also the geographical origin and relevant dietary habits of the subjects (96, 98–100). The most frequently employed experimental designs were represented by cross-sectional and cohort studies, and the duration of the observation period was broadly different among studies, lasting between 1 month to 7 years. Food intake assessment related to the general diet was a common determinant, with the exception of 5 studies.

##### Experimental Methodologies

The analyzed studies primarily used 16S rRNA based metagenomics (*n* = 9), followed by qPCR (*n* = 3) and YIF-SCAN qRT-PCR (*n* = 1) on fecal samples. Different combinations of variable regions of 16S rRNA gene were analyzed by NGS, with V3–V4 and V4 as the most frequent, followed by V1–V4, V1–V9, V3–V5, and V6–V8 (Supplementary Table 1). In addition, 3 studies applied other molecular techniques such as T-RFLP, RAPD-PCR, and DGGE. Finally, 1 of the studies applied the *Streptococcus bovis*/*Streptococcus equinus* complex (SBSEC)-specific PCR assay on colonies isolated on selective media, with primers targeting the 16S rDNA and groEL genes (89).

##### Association With Gut Microbiota Composition

Overall, the association of fermented food consumption with gut microbiota composition varied among the studies, with a common attribute being the higher abundance of Firmicutes, Bacteroidetes, and Proteobacteria in individuals characterized by frequent consumption of fermented foods (with some exceptions). At the genus level, higher abundance of lactobacilli was often linked to the consumption of fermented foods, while in some cases increased levels of bifidobacteria were also observed (91, 92, 96, 99). It is worth noting that in the study comparing healthy adults with diseased subjects suffering from MDD, the intake of fermented milk resulted in an increased number of *Bifidobacterium* only in MDD patients, who had a lower baseline level of this microbial group with respect to controls. However, some MDD patients included in the study were receiving probiotic supplements, thus limiting the interpretation of the results (92).

##### Colonization

Colonization ability was analyzed in 4 studies. More specifically, the overall fecal carriage rate of the foodborne microbe *Streptococcus infantarius* subsp. *infantarius* (Sii) in consumers of local dairy foods was similar to that of non-consumers, but significantly higher prevalence was found in consumers of artisanal butter, as well as in persons handling livestock and livestock primary products (89). The study by van de Pol et al. observed that *Methanobrevibacter smithii*, which was also detected in the milk consumed by the subjects, could be recovered in the feces of almost all of the recruited children (78.2%), indicating that consumption of multiple different dairy products might be associated with increased presence of *M. smithii* in the gut (102). A more complex study analyzed samples collected from infants' gut, as well as from the gut and breast milk of their mothers, revealing that the LAB species identified in dairy foods consumed by the mothers were the same as those present in their gut microbiota. Moreover, the study also revealed vertical transfer of intestinal LAB species from the mother's gut to the milk, and through the milk to the infant's gut, providing indirect additional evidence of the colonization potential of these microbes (97). Finally, the yogurt starter *L. delbrueckii* ssp. *bulgaricus* was detected in 73% of the fecal samples from fermented milk consumers with a consumption frequency of at least 200 g/day in a period of 2.5 years (94).

#### Probiotic Foods

Three of the 20 selected observational articles considered fermented foods containing probiotics, namely LcS (*n* = 2) (104, 105); or combinations of probiotic strains belonging to *Bifidobacterium* spp., and/or *Lactobacillus* spp. (*n* = 1) (106) (Supplementary Table 1).

##### Food Matrices and Study Design

The 2 studies considering diverse commercial fermented foods and fermented milk containing the probiotic strain LcS were carried out on healthy adults and older individuals of both genders. The duration ranged from 1 to 3 months and the assessment was based on intake frequency of fermented foods in relation to the effect on gut microbiota composition. The study considering milk and yogurt containing *Bifidobacterium* spp. and/or *Lactobacillus* spp. probiotic strains (106) was carried out on healthy adults of both genders and lifestyle analysis was included. However, the strains added to the fermented products were not specified in this study. All 3 articles studied the association between consumption of fermented foods and gut microbiota composition, and also evaluated the colonization potential of the probiotic strains.

##### Experimental Methodologies

Two articles (104, 105) used YIF-SCAN qRT-PCR (with group or species-specific primers) to detect changes in specific gut bacterial groups, while one of them focused on the total microbiome as determined by 16S rRNA based metagenomics performed on V3–V4 variable regions of 16S rRNA gene (Supplementary Table 1) (106).

##### Association With Gut Microbiota Composition

The analysis demonstrated an increase in lactobacilli and a positive association with total bifidobacteria upon consistent consumption of fermented foods containing probiotic strains. In particular, frequent consumption of LcS-containing fermented milk (6–7 days/week), was associated with an increased presence of total lactobacilli in the gut microbiota (104). Besides an increase in lactobacilli, in the study of Shima et al. frequent consumption (>3 days/week) of LcS-containing fermented milk positively correlated with the fecal counts of *Bifidobacterium* and total *Lactobacillus* (105). Unlike the two beforementioned studies, in the study of Redondo-Useros et al., comparison between consumption habits (probiotic fermented milk consumers vs. non-consumers), revealed no difference in the lactobacilli taxa, while the abundance of bifidobacteria was increased at all taxonomic levels in probiotic fermented milk consumers (106).

##### Colonization

Concerning the ability to colonize the human gut, the only direct evidence was provided by Shima et al. who reported LcS RNA in the stool samples during the study period (105), whereas the other two articles provided only indirect evidence of this effect. In particular, an increase in the *Lacticaseibacillus casei* subgroup was reported following consumption of probiotic LcS -containing fermented milk (104), while the DNA of *Bifidobacterium* spp. and/or *Lactobacillus* spp. could be detected in the fecal samples of individuals consuming milk and yogurt with the added combination of strains belonging to these genera (106), suggesting a possible colonization ability displayed by probiotics.

## Discussion

The work presented in this systematic review was performed with the purpose of providing a knowledge base to be used as reference for further experimental work addressing the contribution of fermented foods to the health-promoting effects of dietary components. To this aim, we considered both human intervention and observational studies published up to March 1, 2021. Given the relevance and complexity of the intervention studies, a comparative analysis of their main outcomes is summarized in Table 7. Indeed, as expected, the contribution of observational studies to the understanding of the food-gut microbial flow is more difficult to recognize, either because the diet often includes uncharacterized, home-made food items, or because food consumption for each dietary item is mostly inferred from FFQs rather than being experimentally controlled during the study. Actually, the observational studies included in our analysis presented a higher degree of variability, which impaired to draw sound conclusions related to the recurrent microbial groups mainly associated with fermented food consumption, except for lactobacilli and bifidobacteria, followed in some cases by Firmicutes, Bacteroidetes, and Proteobacteria.

**Table 7:** Summary of the comparative analysis of the intervention studies included in this review.

**Food**		***N*** **studies**	**Subjects**				**Study design**			**Intervention**	**Food intake evaluation**	**Probiotic amount**	**Cohort dimension**		**Sequencing strategy**		**Colonization**				**Recurrent gut microbial groups mainly affected**	**Bacteria able to colonize**
			**Healthy**	**Diseased**	**Children**	**Elderly**	**Randomized**	**Before/after**	**Cross-over**	**Duration**		**CFU/day**	**Large studies *N* > 40**	**Pilot studies *N* < 40**	**NGS**	**Other**	**Species-level**	**Strain-level**	**Viability**	**Persistence**		
Probiotics	Traditional	11	6	5	0	0	6	3	2	5 days-24 weeks	5	nd	5	6	7	4	2	1	1	1	Increased L*actobacillus* (*n* = 2)	*B. mongoliense* BMONG18, *P. acidilactici, Staphylococcus, Lc. lactis, Leuc. mesenteroides*
	LcS	13	5	2	3	3	7	5	1	2–24 weeks	2	6.5 × 10^9^-1 × 10^11^	6	7	4	9	0	8	3	6	Increased *Lactobacillus* (*n*= 4) and *Bifidobacteria* (*n* = 5). Decreased *Enterobacteriaceae* (*n* = 4)	LcS
	Other *Lactobacilli*	9	7	1	1	0	3	4	2	1–10 weeks	0	1 × 10^8^-1 × 10^11^	1	8	3	6	5	4	3	7	Increased *Lactobacillus* or LAB (*n* = 6). *Bifidobacterium* divergent results (*n* = 3)	*L*^a^. *paracasei* subsp. *paracasei* CNCM I-1518; *L* ^a^. *paracasei* subsp. *paracasei* CNCM I-3689; *L* ^a^. *rhamnosus* CNCM I-3690; *L. johnsonii* 456; *L. johnsonii* La1; LGG; *L* ^a^. *casei* DN-114 001; *L* ^a^. *paracasei* A
*Bifidobacterium*	12	6	5	0	1	6	4	2	1–8 weeks	2	1 × 10^8^-1 × 10^10^	3	9	6	6	4	1	1	4	Increased *Bifidobacterium* (*n* = 7)	*B. animalis* subsp. lactis; *B. animalis* subsp. *lactis* CNCM I-2494; *B. animalis* subsp. *lactis* DN-173 010 (CNCM I-2494)	
	Synbiotics	5	4	1	0	0	4	1	0	3–12 weeks	1	2 × 10^7^-1 × 10^11^	1	4	2	3	0	2	1	2	Divergent results	*L. acidophilus* La-5; *B. animalis* subsp. *lactis* BB-12; LGG
Total		50	28	14	4	4	26	17	7		10		16	34	22	28	11	16	9	20		

a *Recently renamed Lacticaseibacillus*.

On the contrary, the results of the interventions with traditional or probiotic-added fermented foods were more consistent as a whole, suggesting an impact of the foodborne microbial component on gut microbiota composition, especially for what concerns LAB species and corresponding strains and, to a lesser extent, for bifidobacteria (Table 7). These observations indicate that unselected, environmentally-derived foodborne bacteria embedded within a food matrix are able to survive the adverse conditions of the upper gut, such as low pH and high concentrations of bile salts, to eventually merge with the complex microbial community of the lower gut, where they can find increased opportunities to interact with resident gut communities due to the particularly crowded microbial environment (107). The group of studies employing fermented foods added with well-characterized probiotics, such as the LcS strain of *Lacticaseibacillus casei* which is present at high titers in the commercial drink named Yakult, yielded some attractive results. The presence of LcS bacteria in the gut following supplementation was in fact accompanied by parallel increase in the bifidobacteria population. This suggests that trophic interactions and cross-feeding mechanisms between ingested LcS and resident commensal gut bacteria may have occurred, possibly due to secreted metabolites acting both as growth-promoters toward other resident bacteria, and as essential factors in the health-promoting effects of probiotics (14).

The colonization capacity of foodborne/probiotic microorganisms in the gut environment was tested in only half of the intervention studies analyzed in this work at the species or at the strain level (Table 7). Only few studies provided direct evidence of colonization, by molecular typing of species/strains from live bacteria isolated from fecal samples. More often, the presence of specific foodborne species/strains in the gut was inferred through detection of specific nucleic acid sequences (DNA or, more rarely, RNA) which do not necessarily reflect the presence of viable bacterial cells.

The analysis of colonization persistence in the gut, intended as the presence of the ingested strain(s) following post-intervention washout, is another important indicator that was additionally determined in a subset of the selected intervention studies (Table 7). On the contrary, persistence was never analyzed in observational studies since it is not measurable in this kind of experimental design. The overall results indicate that colonization by foodborne bacteria is a transient condition in most cases. Indeed, resident microbial communities in the human gut have been shown to display a generalized resilience to dietary interventions, rapidly reverting to the baseline structure (16). This points to the need for regular intake of fermented foods to sustain high titers of foodborne species/strains in the host intestine. It is worth noting that the results of colonization analysis were related to the fecal microbial community, due to the ease of collecting stool from humans, but future research should also be addressed to upper gut sites, since the small bowel microbiota is becoming more recognized for mediating host-microbe interactions.

Even though the overall results of the intervention studies analyzed in our review indicate that foodborne microbes display some basic probiotic features, including, in some cases, transient gut colonization capacity, the postulated health-promoting effects of fermented food consumption are still controversial. As discussed in more detail below, this observation strongly supports the need for better standardization of study protocols to prevent conflicting results from hampering progress in the field. Indeed, about 30% of the intervention studies reported no changes in gut microbiota composition following supplementation. Such lack of consistency in the experimental datasets represents a major weak point that needs to be addressed when designing future studies. The primary aspect emerging from our analysis is the broad heterogeneity in the study designs and analysis tools among the different works, which makes it very difficult to draw unequivocal conclusions from their comparison. The complexity of the human gut microbiome is further exacerbated by a broad inter-individual variability in microbiota composition, which can also in turn affect colonization/persistence of supplemented microorganisms (16) acting as an additional confounder. In the absence of a “reference healthy microbiome,” randomized cross-over studies allowing to compare pre- and post-supplementation data from the same subject, should be preferred over placebo-controlled or untreated control trials, as they can better highlight the effect of supplementation over the individual baseline microbiota while increasing statistical power. However, randomized, double-blind, placebo-controlled experimental designs were applied in about 50% of the analyzed intervention studies, while randomized cross-over trials were employed very rarely (Table 7). An alternative strategy described in some studies to avoid interference by inter-individual variability is stratification/clustering of the subjects on the basis of their individual baseline microbiota. This can partially correct some confounders and did allow to detect significant differences that would have otherwise been unnoticed.

The amounts of administered foodborne microbes, the duration of the intervention and the number of participants included in each cohort were also highly variable parameters emerging from our analysis. In the interventions with probiotic fermented foods, the amount of probiotic strains usually ranged between 2 × 10^7^ and 1 × 10^11^ CFU/day (Table 7), in line with a typical microbial exposure from a fermented food-containing diet normally ranging between 1 × 10^8^ and 1 × 10^12^ CFU/day (14). However, very few studies compared the effects of different dosages, and the rationale for choosing a specific one was not specified. This also impaired to identify possible dose-response effects of the ingested microbial component on host gut microbiome perturbations, which is essential to support causal relationships. The amount of supplemented probiotics should always be assessed, and the individual response to dietary interventions or probiotic supplementation should also be taken into account (16).

Duration of the interventions also varied widely among the analyzed studies, ranging between 5 days and 24 weeks for traditional fermented foods, and between 1 and 4 weeks in the majority of probiotic interventions, with the exception of some studies employing LcS where supplementation lasted for 24 weeks (Table 7). The reasons for choosing a specific duration for the interventions was not usually explained by the authors also in this case. We believe that the minimum time span which can induce changes in gut microbiota composition should be determined for each specific fermented food in future investigations.

About 70% of the intervention trials were presented as “pilot studies” involving reduced sample size (Table 7), which leads to lower statistical power and therefore partially undermines the reliability of the results. Moreover, the majority of studies were performed on healthy subjects and only few trials were conducted on diseased patients or elderly subjects (Table 7). It should be noticed that the reported effects of fermented foods on a well-balanced microbiota which is typical of the healthy status, cannot be expected to occur in diseased conditions resulting in intestinal dysbiosis. Therefore, until further experimental results are available, the conclusions from these trials are applicable only to the healthy portion of the population, while it would be extremely important to design effective intervention strategies that can beneficially affect an imbalanced and disease-associated microbiota.

The interaction between foodborne and gut microbes can be qualitatively and quantitatively modified by the concurrent effect of environmental factors, especially those related to the overall diet, which therefore deserves specific attention. With the exception of observational studies, which considered multiple and diverse fermented foods, most of the food matrices employed in the intervention studies were dairies, also representing the most common dietary vehicle for probiotic strains. Accurate assessment of dietary intake was rarely considered in the intervention trials analyzed in this review (Table 7). On the other hand, even if the great majority of observational studies reported food intake data, this aspect was rarely discussed in-depth. We therefore suggest inclusion of food intake assessment in all studies addressing the effects of specific dietary supplementations on gut microbiomes, as it is key to the understanding of their possible interactions with other dietary components often contributing to a great extent to the observed outcomes. Moreover, food intake assessment should be evaluated through optimized questionnaires clearly identifying the contribution of fermented foods to the whole diet. In particular, the extent to which existing FFQs provide direct or indirect data on the intake of live microbes must be determined (108).

A final consideration relates to the different methodological approaches employed for microbiota analysis in the described studies. The advent of high throughput -omic techniques, such as 16S rRNA gene-based NGS and whole genome shotgun metagenomics, have paved the way to a more comprehensive understanding of the changes occurring within complex microbial communities, that can now be detected at the species or genus level including under-represented taxa. Despite the high efficacy of these approaches, they were employed in <50% of the published reports analyzed in this systematic review (Table 7), while the remaining studies applied techniques such as qPCR and/or FISH, DGGE. NGS can provide a wide view of the microbiota as it covers the whole microbial community, while qPCR or FISH cover specific microbial groups. However, these latter methods have the advantage of providing real quantification, and FISH allows to discriminate between live and death bacteria. The most common 16S rRNA based metagenomics approach for microbial community characterization is generally based on the V4, V3–V4 or V4–V5 regions of the 16S rRNA gene (109), with the V4 region recommended as the gold standard for profiling of human gut microbiome (110). Among the different studies reported in the present systematic review, the V3–V4 region was the most frequently analyzed by NGS, followed by the V4.

Concerning the adherence to FAIR principles, the extent to which raw sequencing data were deposited to public available databases was very low, since only 10 studies out of 70 reported this information. The principal repositories used were EMBL-EBI, NCBI, SRA, EGA, MG-RAST, ENA, GenBank.

## Conclusions

In conclusion, the results presented in this systematic review suggest that a complex interplay between food and gut microbiota is indeed occurring, although the possible mechanisms for this interaction, as well as how it can impact human health, still remain a puzzling picture. Moreover, the above-described drawbacks concerning methodological approaches to analyze microbiota composition render extremely difficult to harmonize the resulting datasets.

The availability of advanced computational infrastructures to handle large datasets is providing the scientific community with powerful tools to address research questions of increasing complexity, but we cannot even begin to understand the link between environmental/foodborne microbial diversity and gut microbial assembly unless future studies are properly standardized in terms of study designs, as well as in the experimental procedures for collection, extraction and sample preparation, leading to complete and transparent metadata reporting and appropriate analysis platforms (111–113). Standardization efforts are also necessary to produce FAIR compliant datasets (114), enabling datasharing and in line with the current objectives of Open science (115).

## Data Availability Statement

The original contributions presented in the study are included in the article/Supplementary Material, further inquiries can be directed to the corresponding author/s.

## Author Contributions

CD, MR, and GP contributed to the conception and design of the study. CD, MR, RC, FN, ES, BG, PZ, and ON collected the articles and performed the systematic selection. CD, MR, FN, GP, MD, and MG jointly worked at writing the manuscript. All authors have read and approved the final version of the manuscript.

## Conflict of Interest

The authors declare that the research was conducted in the absence of any commercial or financial relationships that could be construed as a potential conflict of interest.

## Publisher's Note

All claims expressed in this article are solely those of the authors and do not necessarily represent those of their affiliated organizations, or those of the publisher, the editors and the reviewers. Any product that may be evaluated in this article, or claim that may be made by its manufacturer, is not guaranteed or endorsed by the publisher.
