# Supplementary material for: Colonization Ability and Impact on Human Gut Microbiota of Foodborne Microbes From Traditional or Probiotic-Added Fermented Foods: A Systematic Review
Source: Front Nutr. 2021 Jul 29;8:689084. doi: 10.3389/fnut.2021.689084 (PMC8360115; doi:10.3389/fnut.2021.689084)
Supplement: Supplementary file 1 [file Table_1.docx]

**Supplementary Table 1**. Human observational studies focused on consumption of fermented foods, also including probiotics

| **Diet and type of fermented food(s) considered** | **Probiotic(s)** | **Daily amount of fermented food(s)/probiotic(s) and duration of the study** | **Food intake evaluation** | **Study design** | **Subjects main characteristics** | **Association with gut microbiota** | **Gut microbiota analysis methods and microbial groups analyzed** | **Colonization evaluation** | **Methods of analysis for colonization** | **Reference** |
| --- | --- | --- | --- | --- | --- | --- | --- | --- | --- | --- |
| Habitual diet (85 food items, including fermented milk) | No | Fermented milk products: 125 g/day (experimental); 67.5 g/day (control); nd | Yes | Case-control study (n=46, 30 experimental group and 16 control) | Slovakian boys with ASD (autism spectrum disorder); age range 4-9.15 years | Consumption of fermented milk products associated to higher abundances of bacteria, *Clostridia*, *Lactobacillus*, *Blautia*, *Anaerostipes* and *Fusicatenibacter* in ASD group. | 16S rRNA based metagenomics; V1-V9 variable regions of 16S rRNA gene (All bacteria) | No | - | (93) |
| Habitual diet (including fermented plants) | No | Consumers eating fermented plants 3 to 5 times/week, or 1 to 2 times/week and non-consumers eating fermented plants less than once/week or never; 4 weeks | Yes | Cohort study (n=115, 35 consumers and 80 non-consumers) | American healthy adults; age range 19-70 years | Consumers associated with *Bacteroides* spp., *Prevotella* spp., *Enterobacteriaceae*, *Fusobacterium* spp., *Actinomyces* spp., *Clostridium clostridioforme*, *Faecalibacterium prausnitzii*, *Bacteroides uniformis*, *Clostridiales*, and *Lactobacillus* spp. | 16S rRNA based metagenomics; V4 variable region of the 16S rRNA gene (All bacteria) | No | - | (15) |
| Habitual diet, Different diets are assessed (dairies present exclusively in the Indian diet) | No | Habitual diet; 24-h dietary recall; Two 24-h dietary recalls were conducted 2–4 weeks apart once pregnancy was confirmed prior to 12-week gestation. | Yes | Ecological; Cross-sectional study (n=100) | African and Asian healthy women (50 African and 50 Asian); age range 16-35 years | *Succinivibrio* and *Escherichia-Shigella* positively associated with dairy consumption.  *Bifidobacterium* higher in Asian | 16S rRNA based metagenomics; V3-V4 variable regions of the 16S rRNA gene (All bacteria) | No | - | (96) |
| Habitual diet (including dairy products) | No | Dairy (milk, yogurt, ecc.) and cheese intake; ≥2/day, 1/day, >2/week, 1-2/week, <1/week and rarely or never; nd | Yes | Cross-sectional study (n=1000) | French healthy adults (500 men and 500 women); age range 20 to <30 y, 30 to <40 y, 40 to <50 y, 50 to <60 y, and 60 to <70 y old—with 200 subjects per stratum | Milk and dairy consumption (excluding cheese) positively associated with higher abundance of Streptococci and especially with *Streptococcus salivarius*. Cheese consumption associated with higher abundance of *Enterobacteriaceae* and *Lachnospiraceae*. | 16S rRNA based metagenomics; V3–V5 variable regions of the 16S rRNA gene (All bacteria) | No | - | (90) |
| Habitual diet (including spontaneous fermented products) | No | Habitual diet based on fermented products; nd | No | Cross-sectional study (n=29) | Chinese healthy nomads (18 women and 11 men); age range 6-84 years | *Bifidobacterium* and *Collinsella aerofaciens* associated to daily consumption of spontaneous fermented products | 16S rRNA based metagenomics; V3-V4 variable regions of 16S rRNA gene (All bacteria) | No | - | (103) |
| Habitual diet (26 food items, including different fermented dairy products) | No | Dairy products (388.23 g/day); 1 year dietary recall; nd | Yes | Cross-sectional study (n=130) | Spanish healthy adults (38 men and 92 women); age range 58.18 ± 17.10 years | Consumption of natural yogurt directly associated with *Akkermansia* levels, while sweetened yogurt inversely related to *Bacteroides* counts | qPCR with primers covering most relevant gut bacterial groups present | No | - | (95) |
| Habitual diet (including mostly fermented dairy products) | No | Fermented dairy products: 1–2 days per month; 1–2 days per week; 3–5 days per week; 6–7 days per week and unknown frequency; nd | Yes | Cross-sectional study (n=385) | Ivorian healthy adults (men 37.1%, women 62.9%); age range 18-30 years | *Streptococcus infantarius* subsp. *infantarius* (Sii) significantly higher in consumers of artisanal butter. Overall faecal carriage rate of Sii in consumers of local dairy foods similar to that of non-consumers | SBSEC-specific PCR assay targeting 16S rRNA and groEL genes on isolated colonies | Yes | SBSEC-specific PCR assay | (89) |
| Habitual diet | No | Habitual diet: lifestyle and dietary recall from 34 weeks of gestation until 6–7 years of age | Yes | Cohort study (n= 472) | Dutch healthy children; age range 6–10 years | Consumption of organic yogurt and of organic milk positively associated with the presence of *Methanobrevibacter smithii*. Seventy-eight percent of children colonized by *M. smithii*, and 8% by *Methanosphaera stadtmanae* | qPCR with strain-specific primers for *M. smithii* and *M. stadtmanae* (previously used in another study) | Yes | qPCR with species-specific primers for *M. smithii* and *M. stadtmanae* | (102) |
| Habitual diet (120 food items, including dairy products eg. milk, cheese and yogurt) | No | Habitual diet; 6 months dietary recall | Yes | Cohort study (n=37) | Australian healthy children (9 boys and 16 girls); age range 2-3 years | Dairy intake negatively associated with relative abundance of *Bacteriodetes* phylum. Relative abundance of the genus *Streptococcus* positively associated with yoghurt and dairy intake | 16S rRNA based metagenomics; V6-V8 variable regions of 16S rRNA gene (All bacteria) | No | - | (88) |
| Homemade fermented foods (including vegetables and dairies) | No | nd | No | Cohort study (n=7) | American healthy adults members of a local fermenter’s club | *Blautia*, *Varibaculum*, *Bacteroides*, *Peptoniphilus*, and *Corynebacterium* associated with fermented food consumption | 16S rRNA based metagenomics; V4 variable region of 16S rRNA gene (All bacteria) | No | - | (101) |
| Habitual diet. Different diets assessed (Urban: limited variety of foods and absence of fruit and vegetables;  Rural: rich in vegetables, fruit and homemade fermented products) | No | nd | Yes | Cross-sectional study (n=18 and n=10) | Saudis (urban) healthy adult men and Bedouins (rural) healthy adults (8 men and 2 women); age range >18 years | *Bacteroidetes* significantly more common in urban Saudis; *Verrucomicrobia* significantly more common in rural Bedouins.  Rural Bedouins had significantly more bacterial genera present in fermented foods | 16S rRNA based metagenomics; V3-V4 variable regions of 16S rRNA gene (All bacteria) | No | - | (100) |
| Fermented milk | No (although some participants received probiotics) | Consumption of fermented milk classified in 7 groups: none, less than once a week, once a week, 2 to 3 times per week, 4 to 6 times per week, once a day and twice or more per day; nd | No | Case-control study (n=57 control and n=43 patients) | Japanese healthy adults (22 men and 35 women); patients with major depressive disorder (MDD) (25 men and 18 women); average age range 39-55 years | Fermented milk consumption associated with *Bifidobacterium* counts only in MDD group | YIF-SCAN; primers targeting major bacterial groups | No | - | (92) |
| Habitual diet. Different diets assessed including 16 food items (Fermented rice noodles- khanomjeen only included in NE diet) | No | Habitual diet; 1 month dietary recall | Yes | Cross-sectional study (n=31 and n=29) | Thai healthy children (16 girls and 15 boys) living in central (CT) and northeastern (NE) region (16 girls and 13 boys); age range 8-11 years | Higher number of *Bifidobacterium* spp., *Enterobacteriaceae* group, *Lactobacillus* group, *Clostridium leptum* and *Clostridium coccoides-Eubacterium rectale, Prevotella* group, *Bacteroides fragilis* group and methanogeni*c* archaea in NE subjects, who more frequently consumed fermented foods | qPCR with speciﬁc primers targeting total bacteria and *Lactobacillus* group, *Clostridium coccoides–Eubacterium rectale* group, *Clostridium leptum* group, *Bacteroides fragilis* group, *Prevotella* group, *Biﬁdobacterium* spp., *Enterobacteriaceae* and methanogens | No | - | (99) |
| Habitual diet | No | Habitual diet; 1 year and 3 months | Yes | Cohort study (n=10) | Middle-aged Japanese (5 men and 5 women) | Changes in *Bifidobacterium*, but not *Lactobacillus*, associated to seasonal variations in fermented milk consumption | 16S rRNA based metagenomics; V1-V4 variable regions of the 16S rRNA gene (All bacteria) | No | - | (91) |
| Habitual diet rich in local fermented foods | No | nd. | No | Cross-sectional study (n=45) | Syrian healthy infants, their mothers and other healthy adults; infants age range 1 month - 2 years, mothers and healthy adults age range 13–35 years | *L. plantarum* dominant species, followed by *L. fermentum*, *L.*^b^ *brevis*, *L.^a^ casei*, *Enterococcus faecium*, *E. faecalis* and *Pediococcus pentosaceus*. Except for *E. faecium* and *E. faecalis*, they were also dominant species in local fermented foods | Culture-dependent isolation followed by RAPD-PCR and partial sequencing of 16S rRNA gene of colonies | Yes | Culture-dependent isolation followed by RAPD-PCR and partial sequencing of 16S rRNA gene of colonies | (97) |
| Habitual diet. Different diets assessed (Farm: home- grown or farm-produced foods; Anthroposophic: organically and/or biodynamically grown food products, as well as fermented vegetables) | No | Habitual diet; lifestyle and dietary recall | No | Cross-sectional study (n=90; anthroposophic children n=23 and 19 referencegroup living in the same area; children living on a farm n=26 and 22 referencegroup living in the same area) | European children from 3 different countries; age range 5-13 years | Anthroposophic children had a significantly higher diversity of microbes in their feces than farm children, who in turn also had lower diversity than the reference groups | T-RFLP (broad-range bacterial primers) | No | - | (98) |
| Habitual diet. Comparison between consumers and non-consumers of yogurt | No | Daily consumption of 200-400 g yogurt; over 2.5 years | Yes | Cohort study (n=51) | French healthy subjects (30 women and 21 men, consumer group (Y) n=30 and non-consumer group (N) n=21); age range: 35–60 years, | Signiﬁcantly lower levels of *Enterobacteriaceae* in Y group. *Lactobacillus delbrueckii* ssp. *bulgaricus* and *L.^a^ casei* more prevalent in Y group | TGGE (V6-V8 variable regions of the 16S rRNA gene) and FISH (8 phylogenetic groups) | Yes | TGGE and sequencing of the excised band | (94) |
| Habitual diet (including fermented milk and fermented milk with probiotic strain *Lacticaseibacillus casei Shirota* (LcS)) | Yes (LcS) | Habitual diet; lifestyle and dietary recall; Frequency of fermented milk consumption and frequency of fermented milk with probiotic strain LcS: <3 times/week and ≥3 times/week | Yes | Cross-sectional study (n=366) | Japanese healthy adults (185 men and 181 women); average age 40.0±11.0 years | Consumption of LcS-containing fermented milk associated with higher counts of *Lactobacillus*, but lower species diversity. The counts of LcS in stool positively correlated with the counts of *Bifidobacterium* | YIF-SCAN; primers targeting 7 bacterial group, 8 genera, 1 family, 6 *Lactobacillus* subgroups and 5 species which cover 71.3% of total bacterial counts in stool | Yes | qPCR with specific primers for LcS strain and total bacteria counts | (105) |
| Probiotic fermented milk (PFM) and yogurt | Yes (*Bifidobacterium* spp, or *Lactobacillus* spp) | Habitual diet; 1 year lifestyle and dietary recall; For PFM consumers: (a) Low PFM (>0 to <2 products/week; (b) Medium PFM (≥2 to 4 products/week); (c) High PFM (≥5 products/week; PFM-NC: (a) Non-consumers (Y-NC) (0 yogurts/week); (b) Low YC (>0 to 2 yogurts/week) (c) Medium YC (3 to 4 yogurts/week) and (d) High YC (>5 yogurts/week); | Yes | Case-control study(n=260) | Spanish healthy adults (134 men and 126 women; 175 non consumers of probiotic fermented milk (PFM-NC) and 85 consumers of probiotic milk (PFM); further non-consumers of PFM were divided to yogurt consumers 135 and yogurt non-consumers 40); age range 25-50 years | PFM intake increased levels of ingested bacteria. *Actinobacteria*, *Bifidobacteriaceae*, and *Bifidobacterium* associated to the consumption of *Lactobacillus* containing PFM, usually containing *L.*^a^ *casei* strains | 16S rRNA based metagenomics; V3–V4 variable regions of the 16S rRNA gene (All bacteria) | Yes | Inferred from NGS data | (106) |
| Habitual diet (including fermented dairy products containing *Lacticaseibacillus casei* *Shirota* (LcS)) | Yes (LcS) | Habitual diet; Frequency of ingestion of fermented commercial products containing LcS (9x10^8^-4x10^10^ CFU/bottle): a bottle of the product 0–2 days/week, 3–5 days/week, or 6–7 days/week; 1 month assessment | Yes | Cross-sectional study (n=338) | Older healthy Japanese (140 men and 198 women); age range 65-95 years | Total number of *Lactobacillus*, *Lactobacillus* spp. and *Lacticaseibacillus casei* subgroup increased with frequent consumption of LcS fermented milk | YIF-SCAN targeting *Lactobacillus,Bifidobacterium*, *Pseudomonas*, *Staphylococcus*, *Clostridium difficile, Clostridium perfringens*, *Atopobium* cluster, *Bacteroides fragilis* group, *Clostridium coccoides* group, *Clostridium leptum* subgroup, *Prevotella*, *Enterobacteriaceae*, *Enterococcus*, *Streptococcus*)  ; qPCR using primers targeting V1-V2 variable regions of the 16S rRNA gene | Yes | YIF-SCAN with diverse specific primers | (104) |

^a^ Recently renamed *Lacticaseibacillus*

^b^ Recently renamed *Levilactobacillus*
